# Mediterranean versus Red sea corals facing climate change, a transcriptome analysis

**DOI:** 10.1038/srep42405

**Published:** 2017-02-09

**Authors:** Keren Maor-Landaw, Hiba Waldman Ben-Asher, Sarit Karako-Lampert, Mali Salmon-Divon, Fiorella Prada, Erik Caroselli, Stefano Goffredo, Giuseppe Falini, Zvy Dubinsky, Oren Levy

**Affiliations:** 1 The Mina and Everard Goodman Faculty of Life Sciences, Bar Ilan University, Ramat Gan, Israel; 2 Department of Molecular Biology, Ariel University, Ariel, Israel; 3 Marine Science Group, Department of Biological, Geological and Environmental Sciences, Section of Biology, Alma Mater Studiorum–University of Bologna, Bologna, Italy; 4 Dipartimento di Chimica ‘G. Ciamician’, Alma Mater Studiorum Università di Bologna, Bologna, Italy

## Abstract

The anthropogenic increase in atmospheric CO_2_ that drives global warming and ocean acidification raises serious concerns regarding the future of corals, the main carbonate biomineralizers. Here we used transcriptome analysis to study the effect of long-term gradual temperature increase (annual rate), combined with lowered pH values, on a sub-tropical Red Sea coral, *Stylophora pistillata*, and on a temperate Mediterranean symbiotic coral *Balanophyllia europaea*. The gene expression profiles revealed a strong effect of both temperature increase and pH decrease implying for synergism response. The temperate coral, exposed to a twice as high range of seasonal temperature fluctuations than the Red Sea species, faced stress more effectively. The compensatory strategy for coping apparently involves deviating cellular resources into a massive up-regulation of genes in general, and specifically of genes involved in the generation of metabolic energy. Our results imply that sub-lethal, prolonged exposure to stress can stimulate evolutionary increase in stress resilience.

Corals worldwide have been affected by global warming and the accompanying ocean acidification, experiencing reduced skeleton calcification and severe bleaching events that often lead to coral death^1^,^2^. The Mediterranean sea is considered a “biodiversity hotspot”^3^, but is particularly sensitive to climate change^4^,^5^ and to ocean acidification^6^. Mediterranean corals thrive despite a wide annual fluctuation of sea surface temperatures (SSTs), ranging ~13 °C in the Ligurian Sea, compared to sub-tropical corals, facing only a ~6 °C fluctuation in the Red Sea^7^. This demonstrates the adaptability of some scleractinian corals and suggests that understanding the compensatory cellular mechanism of temperate corals is crucial to forecast the overall fate of corals in an era of global warming and ocean acidification.

The effects of global change on corals have been the subject of intensive research in the last decades, but have mainly focused on tropical corals. Here for the first time, we subject a sub-tropical Red Sea coral and a temperate coral from the Mediterranean, to a slow, chronic temperature increase (mimicking the annual rate), together with the low pH values (7.8) expected by the end of the century^1^ in a long-term experiment and analyze the response by ‘in silico’ high-throughput sequencing techniques, aligned with bioinformatics and physiological tools.

The sub-tropical *Stylophora pistillata* (Esper, 1797), a robust^8^, r strategist^9^, scleractinian symbiotic colonial branching, hermatypic coral (Fig. 1C), found across the Indo-Pacific, is a well-studied ‘guinea pig’ coral^10^. The temperate *Balanophyllia europaea* (Risso, 1826), a complex^8^, scleractinian symbiotic solitary oval shaped coral (Fig. 1D), inhabits Mediterranean rocky foreshores to a depth of 50 meters, with dozens of polyps per square meter^11^. Reduced skeletal calcification^12^, higher skeletal porosity^13^ and lower population densities^14^ have been reported with increased seawater temperature and ocean acidification^15^,^16^. To date there are no available genomic nor transcriptomic data available on Mediterranean corals, including on *B. europaea*^17^, whereas an ESTs library was previously constructed for *S. pistillata*^18^.

Our work on the thermotolerant Mediterranean coral *B. europaea* in comparison to S. pistillata contributes to the understanding of how prolonged exposure to sub-lethal stress directs the evolutionary increase in stress resilience.

## Results and Discussion

**Figure 1:**
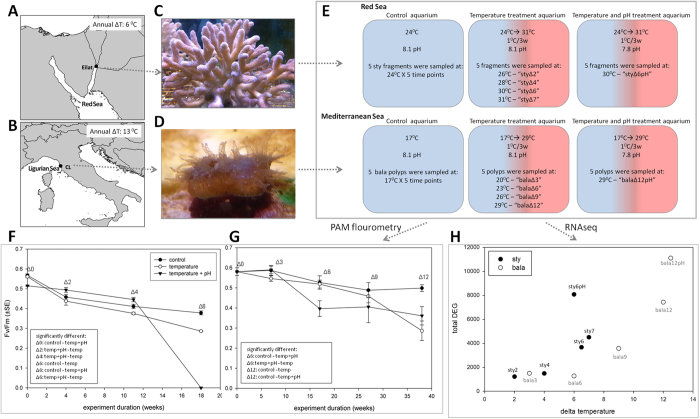
Experimental design, algal maximum quantum yield and numbers of differentially expressed genes. (**A,B**) Sampling locations (Calafuria; CL, Ligurian Sea and Eilat, Red Sea) and their annual delta temperatures. (Made with Natural Earth. Free vector and raster map data @ www.naturalearthdata.com. QGIS Development Team, 2016. QGIS Geographic Information System. Open Source Geospatial Foundation Project. http://www.qgis.org) (**C**) A colony of *S. pistillata* (sty). (**D**) A polyp of *B. europaea* (bala). (**E**) Experimental design. (**F,G**) Algal maximum quantum yield (Fv/Fm) in *S. pistillata* fragments and *B. europaea* polyps, respectively. The numbers shown in the data labels represent the delta temperatures compared to the control in the treatment aquaria. The tables in the left lower corner contain significant differences by one-way ANOVA and post-hoc LSD multiple comparison test (p < 0.05). (**H**) Differentially expressed genes (DEGs) in *S. pistillata* and *B. europaea* by treatment.

*S. pistillata* colonies from the Gulf of Aqaba, Red Sea, and *B. europaea* polyps from Calafuria, Ligurian Sea, north of Italy (Fig. 1A,B), were allocated to Red Sea or Mediterranean conditions, each habitat type comprised three treatments: (1) control with ambient conditions (2) temperature −1 °C increase every three weeks (3) temperature and pH–pH 7.8 with 1 °C increase every three weeks (Fig. 1E). Experimental duration was eight and five months according to the natural SST inclination for the Mediterranean and Red Sea corals, respectively. Coral samples were collected at intervals for RNA extraction and the maximum quantum yield (Fv/Fm) of algal symbionts was calculated as a measure of photosynthetic performance, which is considered as an indicator of thermal stress^19^,^20^,^21^.

For *B. europaea,* Fv/Fm (algal maximum quantum yield) values were higher than those for *S. pistillata*, most notably at the combined treatment of 7.8 pH and 6 °C elevation in comparison to the control (Fig. 1G). This reflects the lower annual change in SST of the sub-tropical habitat (Fig. 1A,B inlet), placing *S. pistillata* as sensitive to heat stress and lower pH in comparison to *B. europaea* (Fig. 1F). Significant change was observed between the temperature and the integrated temperature-pH treatments in some measuring points, implying on a synergistic effect between pH and temperature.

**Figure 2:**
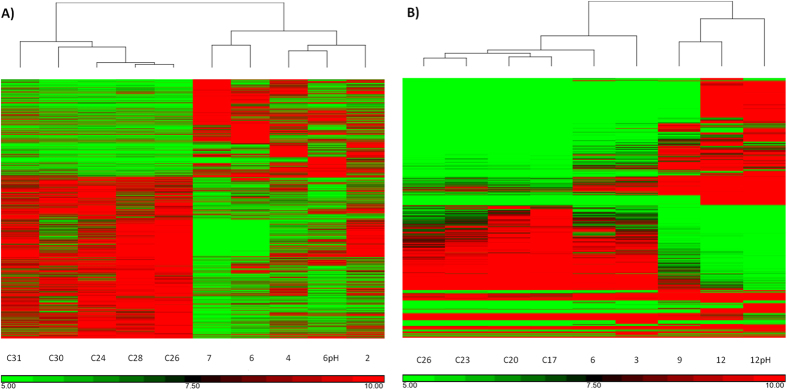
Heat maps of hierarchical clustering of *S. pistillata* (**A**) and *B. europaea* (**B**) samples. 2, 4, 6, 7, 6 pH and 3, 6, 9, 12, 12 pH, stand for the delta in temperature compared to the control, in *S. pistillata* and *B. europaea*, respectively. The Cs stand for the control samples (*S. pistillata*–24 °C, *B. europaea*–17 °C) and the number that follows represents the aquarium temperature at the time point.

*S. pistillata* and *B. europaea* sequenced reads were assembled into transcriptomes of 159,679 and 313,813 open-reading-frame-contigs, respectively and fold changes were calculated for all contigs. Differentially expressed genes (DEGs) were arbitrarily defined as those exceeding a two-fold-change (p-value < 0.05). The first increase in DEGs (Fig. 1H) was seen in *S. pistillata* at 6 °C above control (sty6), but only after 9 °C (bala9) in *B. europaea*. However, in both corals DEG numbers increased with temperature and even more with combined temperature-pH, although the ratio of up- vs. down-regulated genes, showed species differences (Table 1). In *S. pistillata* the percentage of up- and down-regulated genes were similar throughout most of the experiment, but in *B. europaea* the percent of down-regulated genes increased from 3% of total DEGs at the beginning, to 43% by the end of the experiment. With combined temperature-pH treatment, most (71%) of the DEGs were down-regulated in *S. pistillata* but not in *B. europaea*. The clustering in control samples can be seen in three-dimensional space principle components (PC) (Fig. S1) and hierarchical clustering heat maps (Fig. 2). An elevation of only 2 °C was sufficient to change the pattern of gene expression in *S. pistillata* but at least an increase of 9 °C was needed to separate *B. europaea* from the control.

**Figure 3:**
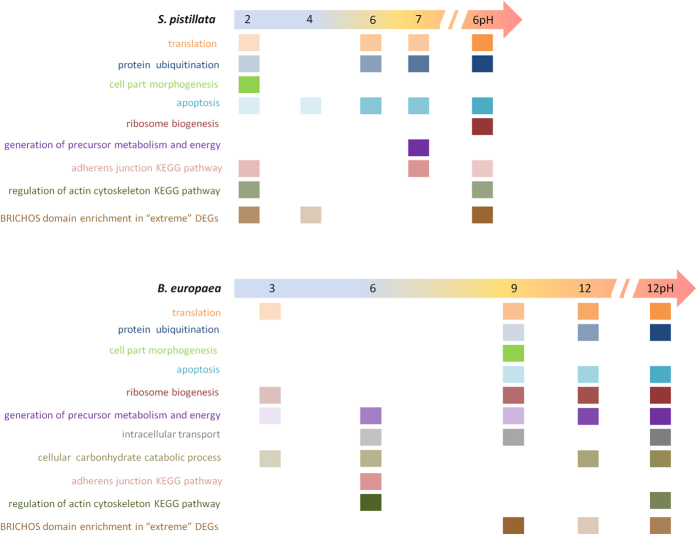
A model summarizing enriched cellular processes in *S. pistillata* and *B. europaea* throughout the experiment. The categories on the left are based on Gene Ontologies and KEGG pathways retrieved David or STRING databases. Treatments giving BRICHOS domain enrichment in “extreme” (at least ten fold change) DEGs. 2, 4, 6, 6 pH, 7 and 3, 6, 9, 12, 12 pH indicate the delta of temperature compared to the control, in *S. pistillata* (sty) and *B. europaea* (bala), respectively. The color intensity gradient of the squares represents the enrichment of the treatment with the GO/pathway.

A model summarizing selected Gene Ontologies (GO) and pathways in *S. pistillata* and *B. europaea* over the course of the experiment shows the up/down regulated cellular processes and implies different cellular responses to stress (Fig. 3). GOs enriched early in *S. pistillata*, including protein ubiquitination and apoptosis, appear only later in the experiment in *B. europaea*. GOs that are related to cell morphological changes could be a manifestation of this heat stress in *S. pistillata*. In contrast, ribosome biogenesis, generation of precursor metabolism and energy, and intracellular transport processes, appear earlier in *B. europaea* than *S. pistillata*, while the process of carbohydrate catabolism is absent from *S. pistillata.* We postulate that the enrichment of metabolic processes (generation of precursor metabolism and energy and cellular carbonhydrate catabolic process) might enable *B. europaea* to postpone temperature-induced protein degradation and cell death.

Enrichment analyses of the specifically temperature-pH DEGs were generated for each coral species (see in Fig. S5 Venn diagram the 5,984 and 4,857 unique temperature-pH genes of *S. pistillata* and *B. europaea*, respectively) and compared to the matching temperature treatment (unique sty6pH to sty6 and unique bala12pH to bala12) (Tables S4 and S5). This analysis revealed that as expected^22^,^23^, transport processes were modified by temperature and pH in both corals, but in *S. pistillata* there was an effect on protein degradation while the response to stress in *B. europaea* involved metabolic processes.

**Figure 4:**
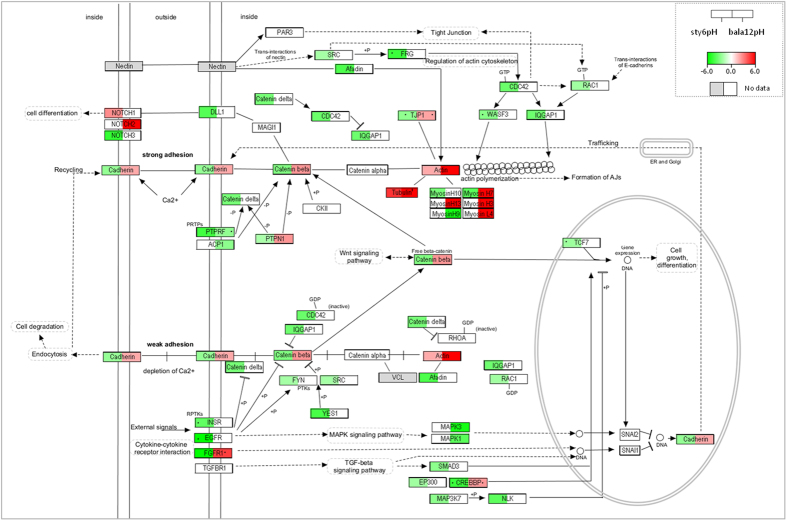
Adherens junction (AJ) pathway and *S. pistillata* +6°C-pH (sty6pH) and *B. europaea* +12 °C-pH (bala12pH) fold changes. Fold changes of sty6pH (left) and bala12pH (right) genes are represented by a color scale. A dot stands for an insignificant contig annotation corresponding to each organism and ref. 1 for an average fold change value of several contig annotations. AJs are mediated through calcium-dependent cadherins linked to the actin cytoskeleton via α-and β-catenins^49^. Chrodate-like classic cadherins were not observed in the cnidarian *Nematostella*^49^, as well as in our studied corals, however, the cadherin genes found belong to the protocadherin subfamily of cadherin superfamily. Regulation of AJs can be by α-catenin interactions with actin-associated proteins (Afadin and VCL), via the small GTPase RAC1 and Cdc42^50^, or alternatively through posttranslational modifications of AJ components. Cadherin-catenin complexes are negatively regulated by tyrosine phosphorylation of β-catenin by receptor tyrosine kinases or cytoplasmic tyrosine kinases; SRC, Fyn and YES1 and positively regulated by casein kinase II (CKII) and protein tyrosine phosphatases (PTPs). Additional regulation is through GTPase-activating protein IQGAP binding to RAC1, which is thought to displace the latter from interacting with β-catenin, thereby freeing β-catenin to interact with α-catenin. Dissociation of β-catenin is likely to render cadherin susceptible to proteasome-mediated degradation or recycling back to the cell surface^50^. WNT signaling acts as a positive regulator, activating transcription factors by stabilizing β-catenin and promoting its binding to TCF (transcription factor3). Various growth factors, including: HGF, FGF, and TGFβ2, regulate cadherin expression indirectly via transcription factor SNAI2/Slug, which eventually inhibits cadherin expression. Transcriptional activation of β-catenin is stimulated via TGF-beta signaling by enhancing the interaction between CREBB-binding protein and SMAD3^51^. Adhesion of neighboring cells can also be mediated via NOTCH and DLL1 or nectin, which results in lateral inhibition of cell differentiation and firm adhesion.

Thirty-nine DEGs, identified from the KEGG database as being associated with the adherens junction (AJ) pathways and regulation of actin cytoskeleton were enriched in *S. pistillata* at +2 °C and in *B. europaea* at +6 °C (Fig. 4). Interestingly, detachment of endoderm cell containing *symbiodinium* is one of the proposed mechanisms of coral bleaching^24^. A combination of 30 °C and pH7.8 down regulated *S. pistillata* β-catenin and δ-catenin as well as cadherin, weakening cell-cell adhesion and facilitating coral bleaching^25^. In contrast, at +6 °C the cadherin-catenin positive regulator casein kinase II (CKII) as well as other AJ elements were up-regulated in *B. europaea* (Fig. 4) with increased temperature and pH.

ScanProsite^26^ domain-prediction analysis of “extreme” DEGs, (changing at least ten fold) revealed an enrichment of proteins (Fisher’s exact test, 2-tail p < 0.05) containing the conserved BRICHOS domain^27^ in *S. pistillata*; +2 °C, +4 °C and especially at +6 °C-pH (up to 380 fold increase), and in *B. europaea* at; +9 °C, +12 °C and +12 °C-pH (Fig. 3). Crystal structure^27^ and functional mutation^28^ studies strongly imply that BRICHOS domain is acting as a chaperone. BRICHOS domain was uncovered in a hydrothermal worm and was hypothesize to contribute to the worm’s evolution-driven unique adaptation to survive under extreme conditions^29^. Clustal Omega alignment^30^ of 238 BRICHOS domain sequences retrieved from 15 coral species transcriptome databases) (http://comparative.reefgenomics.org/datasets.html)^17^ using ScanProsite^26^ (Fig. S7), revealed that corals shared the overall conserved BRICHOS architecture^31^ including the pair of cysteine residues (positions 30 and 105) that probably form a disulfide bridge^31^. Moreover, some residues were specific for corals (for example A in position 7, G-81, N-98). At the present stage we can only speculate whether proteins possessing this domain could play a major role as unique chaperones or fulfill some other yet unknown novel function in the coral response to environmental stress.

This study demonstrates that temperate corals used to higher annual temperature fluctuations than those faced by tropical species, have developed thermotolerance and resistance to pH change. It has been previously shown that daily environmental fluctuations in a physically challenging microhabitat promote thermotolerance in corals^32^,^33^,^34^. Our results are also in agreement with the concepts developed by Nevo^35^, showing, that sub-lethal, prolonged exposure to any stress can stimulates genetic polymorphism and the resulting evolutionary increase in stress resilience. A recent work by Hume *et al*.^36^ argue that positive selection can facilitate adjustment to temperature extremes of coral symbionts in the Persian/Arabic Gulf which endorses these concepts as well.

A deeper understanding of the genomic plasticity across latitudes and marine ecological niches may improve our projections of the ‘winners and losers’ and the future of coral ecosystems under the threat of global change.

## Material and Methods

### Coral collection and maintenance

*Balanophyllia europaea* polyps were collected from Calafuria, in the Ligurian Sea, north of Italy, shipped to Bar-Ilan University, Israel, and placed into an aquarium maintained at 17 °C for several months prior to the experiment. *Stylophora pistillata* colonies were collected while SCUBA diving at a depth of 10 m in the Gulf of Aqaba, Eilat (Red Sea) and maintained at 24 °C at Bar-Ilan University, Israel.

All the corals were acclimated and maintained in 600 L aquariums with circulating artificial seawater (Brightwell Aquatics) under controlled constant conditions of temperature and 35‰ salinity, mimicking the average annual ambient temperatures conditions. The annual cycle of diurnal-dimming light regime was simulated with an Advanced Control Lighting System (ACLS, Sfiligoi, Italy) and HQI (Hydrargyrum quartz iodide) light bulbs (400 W, 14000 Kelvin). The corals were fed once a day with a microvore microdiet (Brightwell Aquatics). Following the acclimation period, the corals were transferred into 300 L aquariums, with the running flow controlled by a computer system, through closed circulation to compensate for salinity fluctuations and water level changes (constant salinity level of 35‰).

### Experimental design

For the Red Sea coral experiments, six *S. pistillata* colonies were placed in each 300 L aquarium: (1) Control aquarium–constant 24 °C (mimicking the average annual ambient temperatures conditions) and ambient pH. (2) Temperature treatment–Increase in temperature from 24 °C to 31 °C at a rate of 1 °C every three weeks (mimicking the annual rate in Eilat) under ambient pH. (3) Temperature and pH treatment −1 °C increase every three weeks from 24 °C to 31 °C while maintaining a pH of 7.8. Two fragments approximately 2 cm long were chopped and sampled from the upper branches of every colony and aquarium, at the beginning of the experiment and a week after the temperatures reached: 26 °C, 28 °C, 30 °C, and 31 °C.

For the Mediterranean coral experiments, *B. europaea* polyps were placed in each of three aquaria: (1) Control aquarium - constant 17 °C (mimicking the average annual ambient temperatures conditions) and ambient pH. (2) Temperature treatment–Increase in temperature from 17 °C to 29 °C at a rate of 1 °C increase every three weeks under ambient pH. (3) Temperature and pH treatment −1 °C increase every three weeks from 17 °C to 29 °C while maintaining a pH of 7.8. Five polyps from every aquarium were sampled, at the beginning of the experiment at 17 °C and then a week after the temperatures reached: 20 °C, 23 °C, 26 °C and 29 °C. The sampled fragments and polyps were snap-frozen in liquid nitrogen and kept at −80 °C for RNA extraction.

### PAM fluorometer

A MAXI imaging Pulse Amplitude Modulation (iPAM) fluorometer (Heinz Waltz GmbH, Germany) was used to evaluate the maximum quantum yield of photosystem II of the algal symbionts of Red Sea *S. pisitillata* and Mediterranean *B. europaea*. Following 30 min of dark adaptation, triplicate measurements of the fluorescence in each aquarium were taken at each time point.

### RNA extractions

RNA was extracted from *B. europaea* polyps using Total RNA kit (A&A Biotechnology), according to manufacturer’s instructions, with the minor modifications that all the centrifugations were cooled and the RNA was extracted using double the recommended amount of Fenzol (in order to cover the entire fragment), and was vortexed during the 5 minute incubation. Following the incubation, the tube was centrifuged 12,000 g, 20 min, in order to reduce algae cell contamination and remove the remains of the unwanted pellet. The final elution volume was 50 μl.

Total RNA was extracted from *S. pistillata* fragments using Trizol (Invitrogen Life Technologies, Carlsbad, CA, USA) according to the methods previously described^37^.

The concentration of the RNA was measured using a NanoDrop spectrophotometer (ND-1000), and the quality (RIN number >8.5) assessed by a Bioanalyzer (Agilent).

### Transcriptome sequencing, library preparation and assembly

Samples of total RNA from *S. pistillata* and *B. europaea* were pooled (into 1 μg each sample) and sent to the IGA Technology Services facility in Udine, Italy for pair-end Hiseq barcoding. Upon arrival at the IGA total RNA of the samples was re-analyzed with an Agilent 2100 Bioanalyzer system (Agilent, Waltham, MA) to ensure that no damage had occurred during shipment. Libraries were prepared using the ‘TruSeq mRNA Sample Prep kit’ (Illumina, San Diego, CA) according to the manufacturer’s instructions. Poly-A mRNA was fragmented (3 min at 94 °C) and every purification step was performed using 1X Agencourt AMPure XP beads.

The final libraries were quantified by using a Qubit 2.0 Fluorometer (Invitrogen, Carlsbad, CA) and quality tested by Agilent 2100 Bioanalyzer High Sensitivity or DNA 1000 assay (Agilent Technologies, Santa Clara, CA). Libraries were then processed with Illumina cBot for cluster generation on the flowcell and sequenced in paired-end mode on HiSeq2500 (Illumina, San Diego, CA), following the manufacturers’ instructions for a rapid run. The CASAVA 1.8.2 version of the Illumina pipeline was used to process raw data for both format conversion and de-multiplexing. The Fastq files have been deposited at SRA database under the accessions: SRP075606 (*Balanophyllia europaea*) and SRP075598 (*Stylophora pistillata*).

Pooled sequences from the 20 sampled treatments were used (10 for each coral) to assemble the *S. pistillata* and the *B. europaea* transcriptomes. Trim Galore software (www.bioinformatics.babraham.ac.uk/projects/trim_galore/) was used for trimmed off sequencing adapters, low quality reads and for quality control. The reads were submitted to Trinity software (version r20131110)^38^ for *de novo* assembly using the default parameters. *S. pistillata* and *B. europaea* library assemblies resulted with 470,497 and 961,667 contigs (transcripts), respectively. Each set of reads from each sample was then aligned to the assembled transcriptome using Bowtie, and RSEM^39^ was next applied in order to get sample-specific abundance estimates. The last two steps were done using the “run_RSEM_align_n_estimate.pl“ script from the Trinity software package using the default parameters. In order to minimize false positive isoforms, all transcripts having FPKM value <1 or isoform percentage values (IsoPct%) <1 were filtered out, and were not included in downstream analysis. 162,784 contigs (N50 = 2088, median contig length = 687) and 546,169 contigs (N50 = 1246, median contig length = 424)) were left after filtering in *S. pistillata and B. europaea* assemblies respectively. Putative coding regions were extracted from the transcriptome assemblies using TransDecoder software (www.transdecoder.sourceforge.net), providing all the CDS and proteins from the assembly. The *S. pistillata* library contained 159,679 open reading frame (ORF)-encoding contigs and the *B. euorpaea,* 313,813. From which 71,096 and 213,664 protein coding genes are for *S. pistillata* and *B. europaea*, respectively. Bowtie (v2.1.0) was again used to map each set of reads from each sample against CDS, FPKM was calculated for expression values.

Annotations were created by blasting the proteome against the *Homo sapiens* database using BLASTP (NCBI). Annotations were also created from the Swissprot and Uniprot50 databases by BLASTP. We applied filtering to the BLASTP results in order to increase the certainty of obtaining true homologs. With regards to the *H. sapiens* annotations, our filtering parameters were set at an e-value threshold of 5 * 10^−5^, >30% alignment identity and >70% query coverage. After annotation filtration, 22,881 annotated contigs remained for *S. pistillata* and 61,908 contigs for *B. europaea*.

### Validation using quantitative real-time PCR

To validate the RNA-seq results, quantitative real-time polymerase chain reaction (qRT-qPCR) assays were performed for four highly expressed selected genes and a comparison made between the results in the treated and control samples. For *S. pistillata*, small heat shock protein and calmodulin genes, which were up- and down-regulated respectively, after 30 °C treatment, were selected. For *B. europaea*, we selected ferritin and nitric oxide synthase, which were up-regulated after 29 °C treatment.

Complementary DNAs were synthesized from 1 μg of total RNA with 1 μl Solaris RNA spike (Thermo-Scientific) using the qScript cDNA synthesis Kit (Quanta Biosciences), according to the manufacturer’s instructions. Specific qRT-PCR primers (Table S6) were designed to amplify 100–200 bp PCR products. Assuming equal RNA loading, the Solaris spike controls are designed to act as a synthetic exogenous control to identify the presence of reaction inhibition thereby circumventing the need for a housekeeping gene^40^,^41^,^42^. Spike-inoculated cDNA aliquots were diluted 1:10 and a 4 μl sample was used in technical triplicates for 10 μL qRT-PCR reactions including 0.5 μl mix of forward and reverse primers, 5 μl of GoTaq qPCR Master Mix (Promega), and 0.5 μl of RNAse free water, for 45 cycles. A melt curve analysis obtained by incubating the reactions for 10 s at 0.5 °C increments between 60 °C and 90 °C was generated for each pair of primers, to test for nonspecific amplification products. The comparative ΔΔCTs method was used, and fold changes were calculated using the 2^−ΔΔCt^ formula to estimate the relative amounts of transcripts in each sample^43^. Ct refers to the cycle at which the fluorescence signal crosses the threshold and by using the solaris spike control^40^,^41^,^42^ we normalized the expression to RNA loading. The MIQE guidelines were taken into account in designing real time profiles and analyzing their results^44^.

Relative expression values for each gene and each biological replicate were calculated by the ratio of treatment relative expression over the average of control relative expression. The results of qPCR and RNA-seq fold change analyses are presented on a log2 scale (Fig. S8). Our results show that there is a correlation between the RNA-seq and the qPCR results, based on log2 fold gene expression (adjusted r^2^ = 0.86; p < 0.0001). The fold changes calculated based on qRT-PCR were in the same direction and consistent with those in the RNA-seq data, thus confirming the RNA-seq results and validating our approach (Fig. S8).

### Bioinformatic analysis

We utilized Partek Genomic suite software (version 6.6, Copyright©2012, Partek Inc., St. Louis, MO, USA) to plot a three-dimensional space principle component analysis and to generate Venn diagrams and hierarchical clustering heat maps for *S. pistillata* and *B. europaea* samples (Heat maps were generated using Euclidean distance as a similarity measure and average linkage).The fold changes were calculated for all contigs using the average of the relevant controls (each coral species by its control samples). An arbitrary cutoff of at least two fold (p-value < 0.05) was chosen to define a differentially expressed gene (DEG).

Functional gene analysis was done by David Bioinformatics Resources 6.7^45^,^46^ and KEGG pathway analysis via STRING 9.0 database^47^, using the *Homo sapiens* orthologs annotations. Retrieving Gene Ontology (GO) biological processes (see core enriched GOs at Figs S2 and S3) enabled an enrichment analysis and analysis of the coral responses to long-term chronic stress.

We utilized a list of Cnidarian environmental stress genes^48^ based on relevant literature to search the Swissprot and Uniprot 50 annotations. This indicated a differential expression between *S. pistillata* and *B. europaea* with respect to known Cnidarian environmental stress genes (Fig. S4) that corresponds to the results of the enrichment analysis.

## Additional Information

**How to cite this article**: Maor-Landaw, K. *et al*. Mediterranean versus Red sea corals facing climate change, a transcriptome analysis. *Sci. Rep.* **7**, 42405; doi: 10.1038/srep42405 (2017).

**Publisher's note:** Springer Nature remains neutral with regard to jurisdictional claims in published maps and institutional affiliations.

## Supplementary Material

Supplementary Information

## Figures and Tables

**Table 1:** Percentage of down-regulated genes out of total differentially expressed genes (DEG) in *S. pistillata* and *B. europaea* treatments (2, 4, 6, 7 and 3, 6, 9, 12; temperature elevation above the control of *S. pistillata* and *B. europaea*, respectively, and pH7.8).

*S. pistillata*	Total DEGs	% ↓	*B. europaea*	Total DEGs	% ↓
2	1219	44.9	3	1495	2.9
4	1487	37.9	6	1274	7.0
6	3668	53.1	9	3572	19.8
7	4504	58.3	12	7425	42.3
6 pH	8072	71.1	12 pH	11102	43.6
