# Supplementary material for: Mediterranean versus Red sea corals facing climate change, a transcriptome analysis
Source: Sci Rep. 2017 Feb 9;7:42405. doi: 10.1038/srep42405 (PMC5299404; doi:10.1038/srep42405)
Supplement: Supplementary Information [file srep42405-s1.doc]

Supplementary Materials for

Mediterranean versus Red sea corals facing climate change, a transcriptome analysis

Keren Maor-Landaw^1^, Hiba Waldman Ben-Asher^1^, Sarit Karako-Lampert^1^, Mali Salmon-Divon^2^, Fiorella Prada^3^, Erik Caroselli^3^, Stefano Goffredo^*3^, Giuseppe Falini^4^, Zvy Dubinsky^1^ and Oren Levy^*1^

^1^ The Mina and Everard Goodman Faculty of Life Sciences, Bar Ilan University, Ramat Gan, Israel.

^2^ Department of Molecular Biology, Ariel University, Ariel, Israel.

^3^ Marine Science Group, Department of Biological, Geological and Environmental Sciences, Section of Biology, Alma Mater Studiorum–University of Bologna, Bologna, Italy.

^4^ Dipartimento di Chimica ‘G. Ciamician,’ Alma Mater Studiorum Universita` di Bologna, Bologna, Italy.

*Corresponding authors – Stefano Goffredo; email: [s.goffredo@unibo.it](mailto:s.goffredo@unibo.it)

and Oren Levy; email: [oren.levy@biu.ac.il](mailto:oren.levy@biu.ac.il)


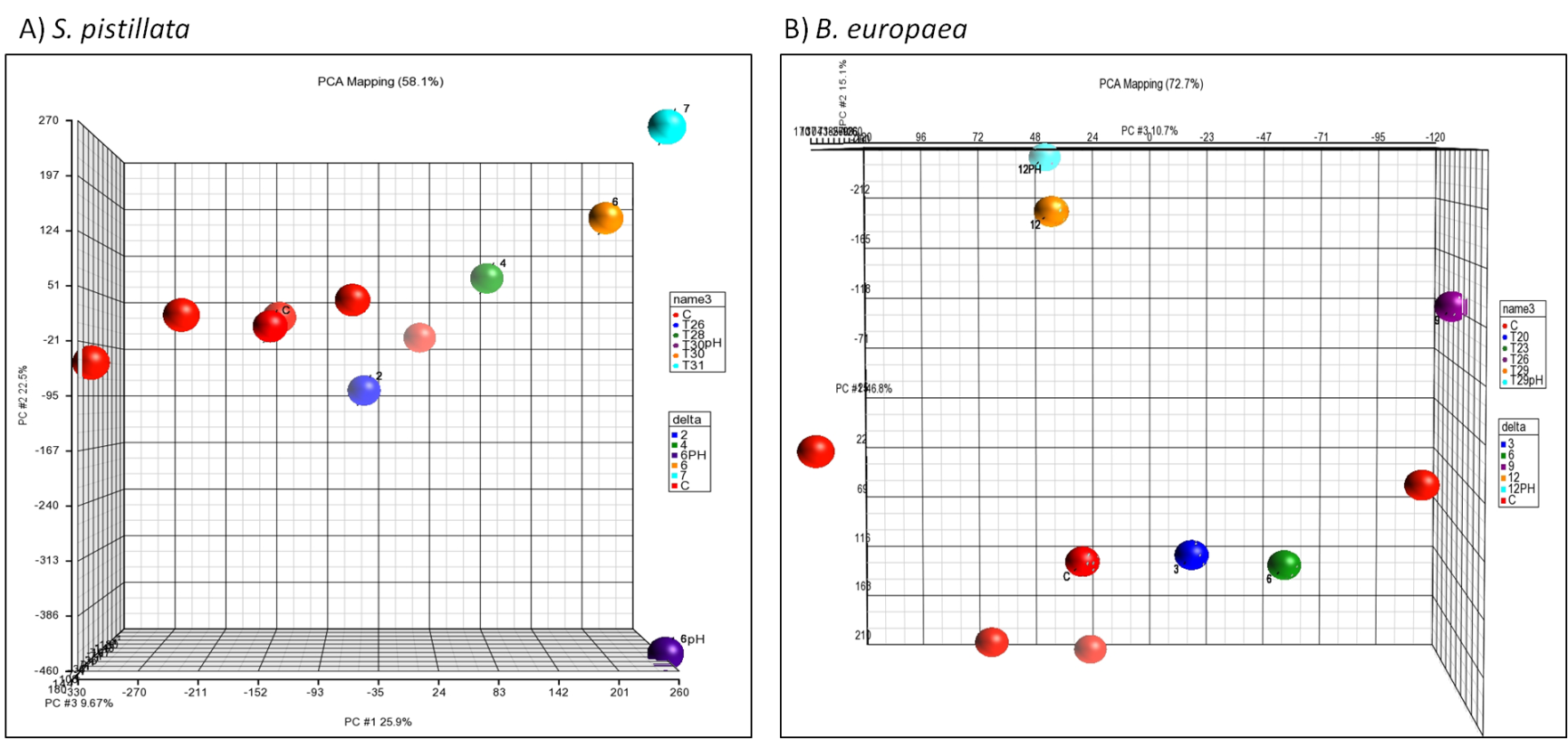


Fig. S1.Principle component analysis (PCA) of *S. pistillata* (A) and of *B. europaea* (B) treatment and control samples. 2, 4, 6, 7, 6pH and 3, 6, 9, 12, 12pH, stand for the delta in temperature compared to the control, in *S. pistillata* and *B. europaea*, respectively. The C stand for the control samples (*S. pistillata* – 24^0^C, *B. europaea* – 17^0^C).


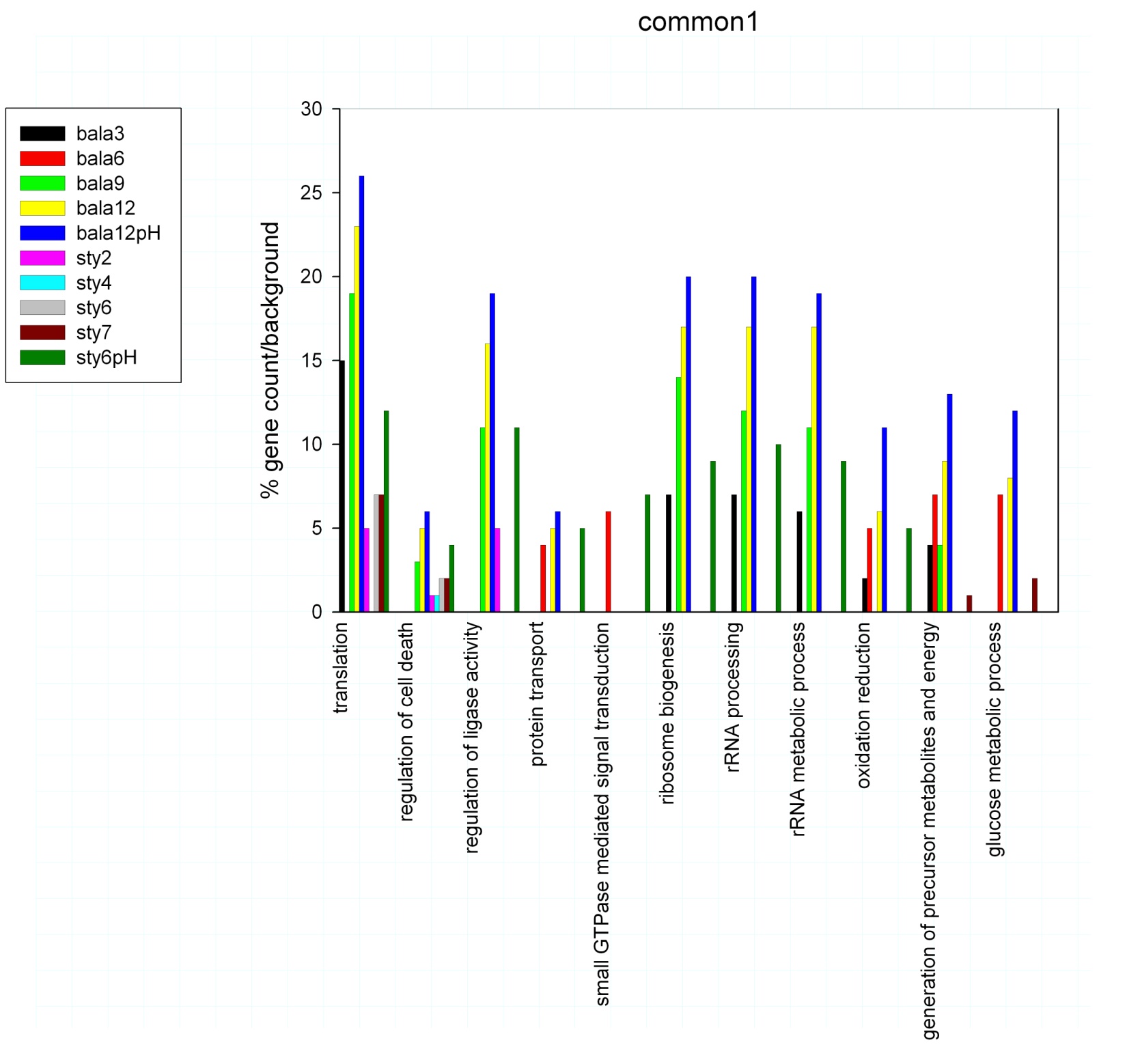


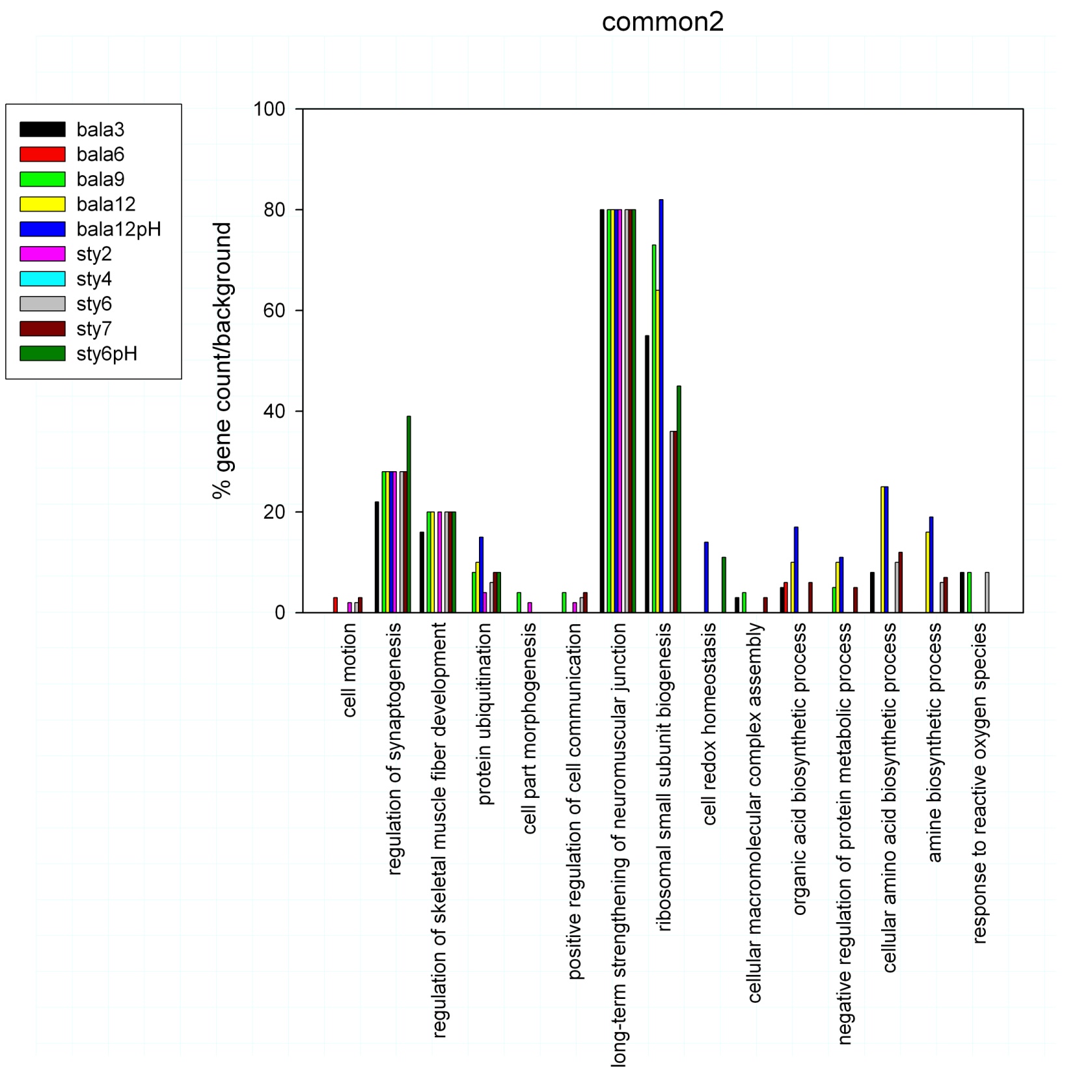


Fig. S2

Enriched selected Gene Ontologies detected in *S. pistillata* and in *B. europaea*. GOs were retrieved from David Bioinformatics Resources 6.7 (pval<0.01) and the data is presented as the percentage of the number of genes in each GO category out of the total genes in the background of the GO.


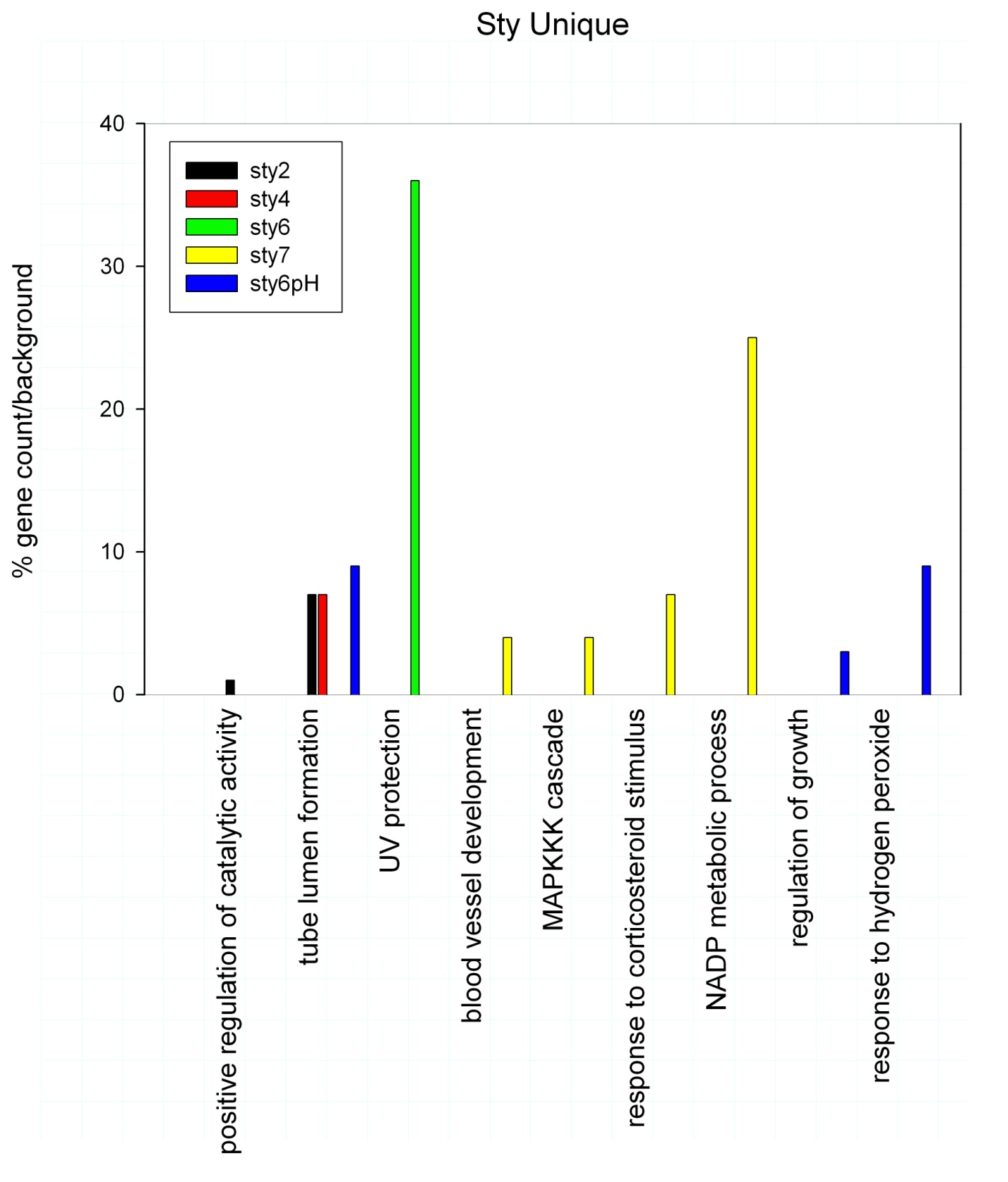


A)


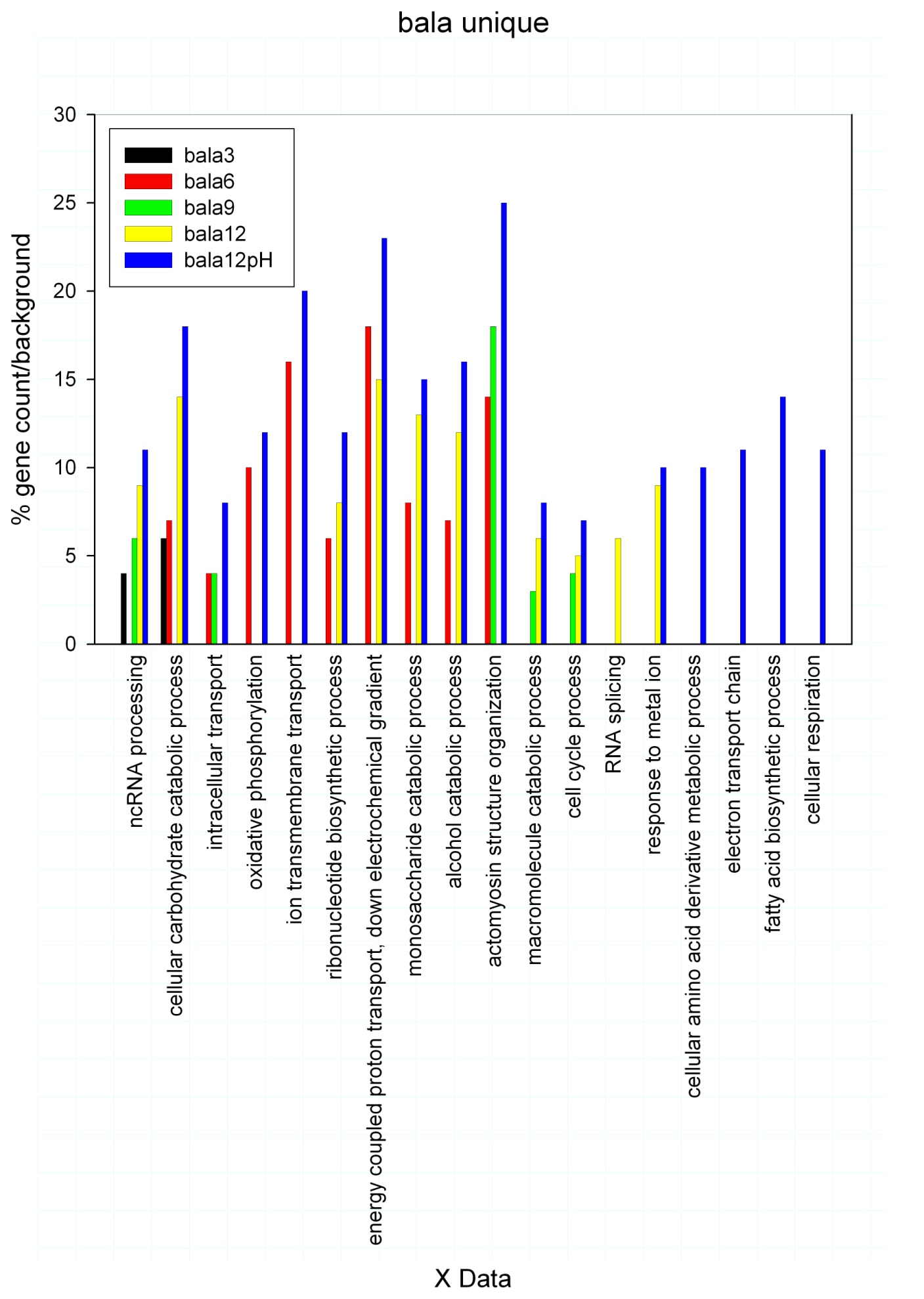


B)

Fig. S3

Enriched selected Gene Ontologies detected uniquely in *S. pistillata* (A) or *B. europaea* (B). GOs were retrieved from David Bioinformatics Resources 6.7 (pval < 0.01). The data is presented as the percentage of the number of genes in each GO category out of the total of genes in the background of the GO.


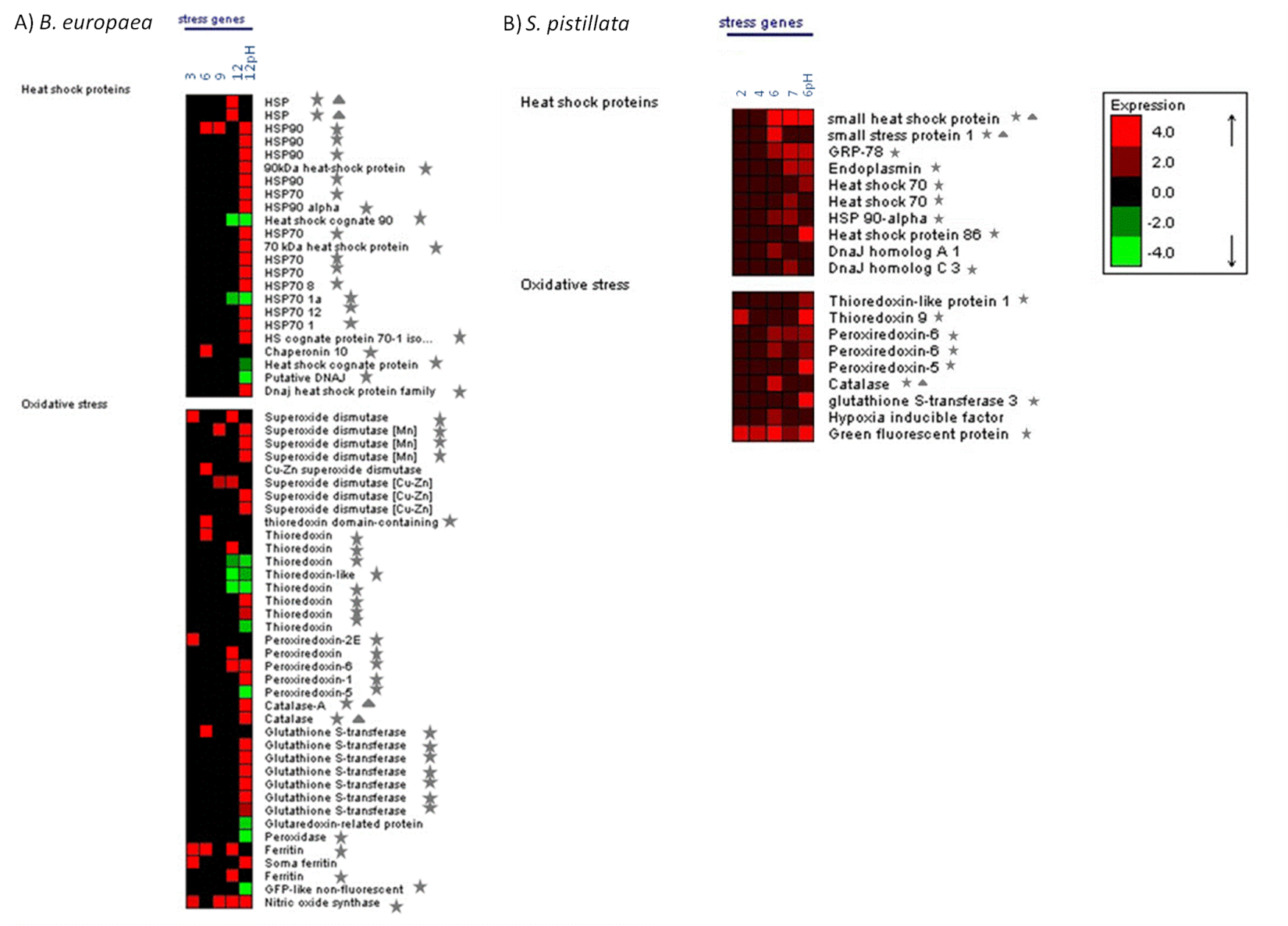


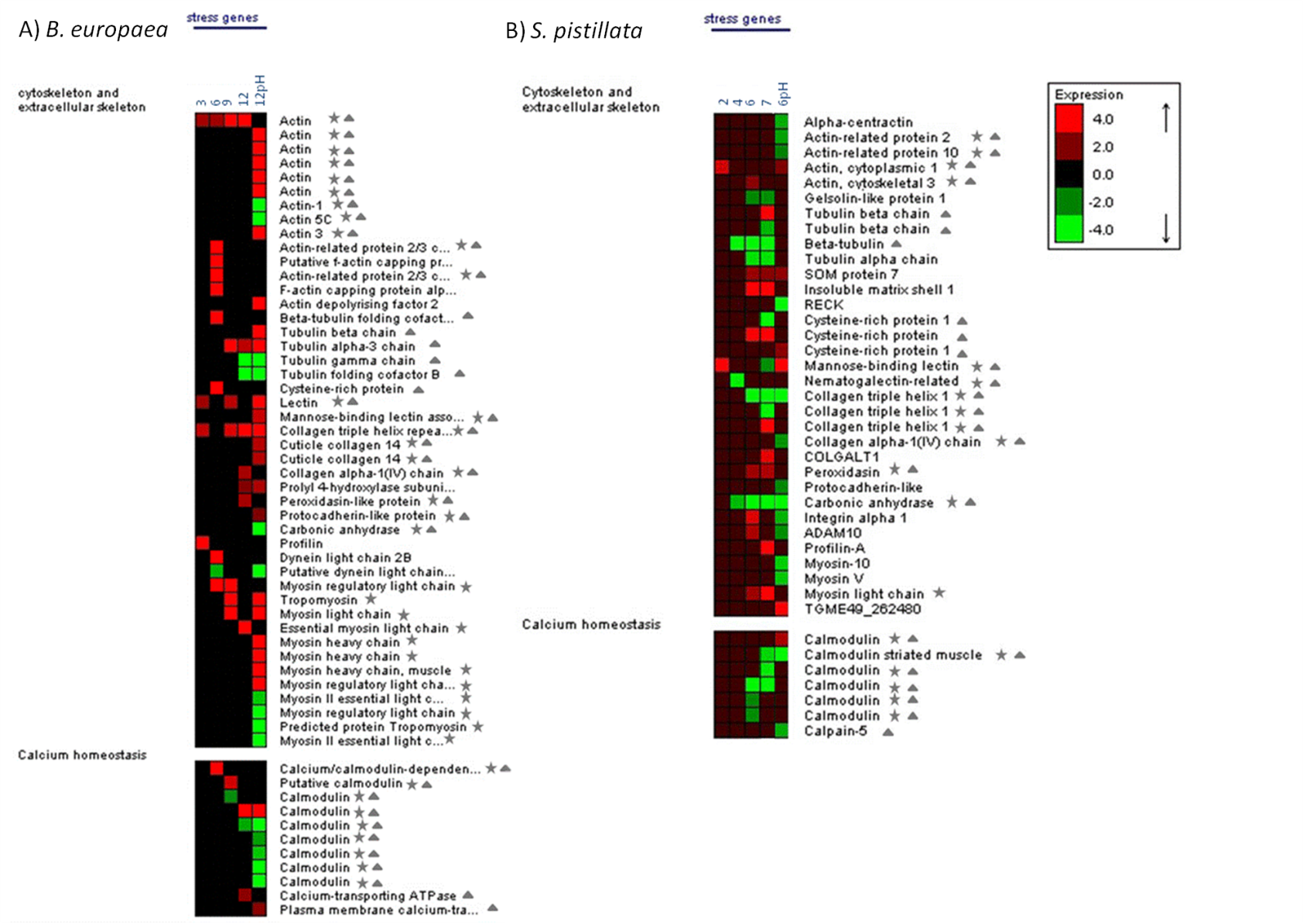


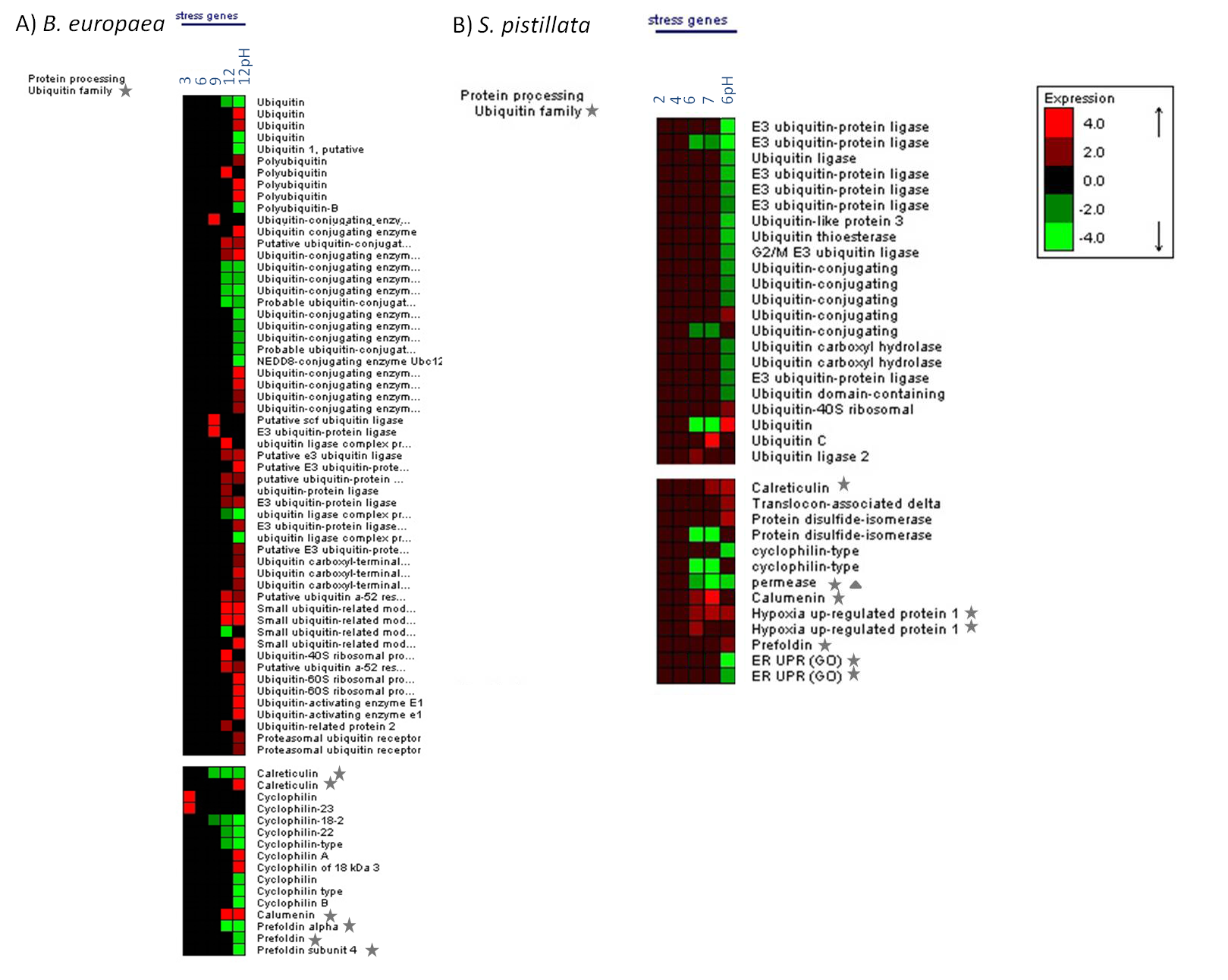


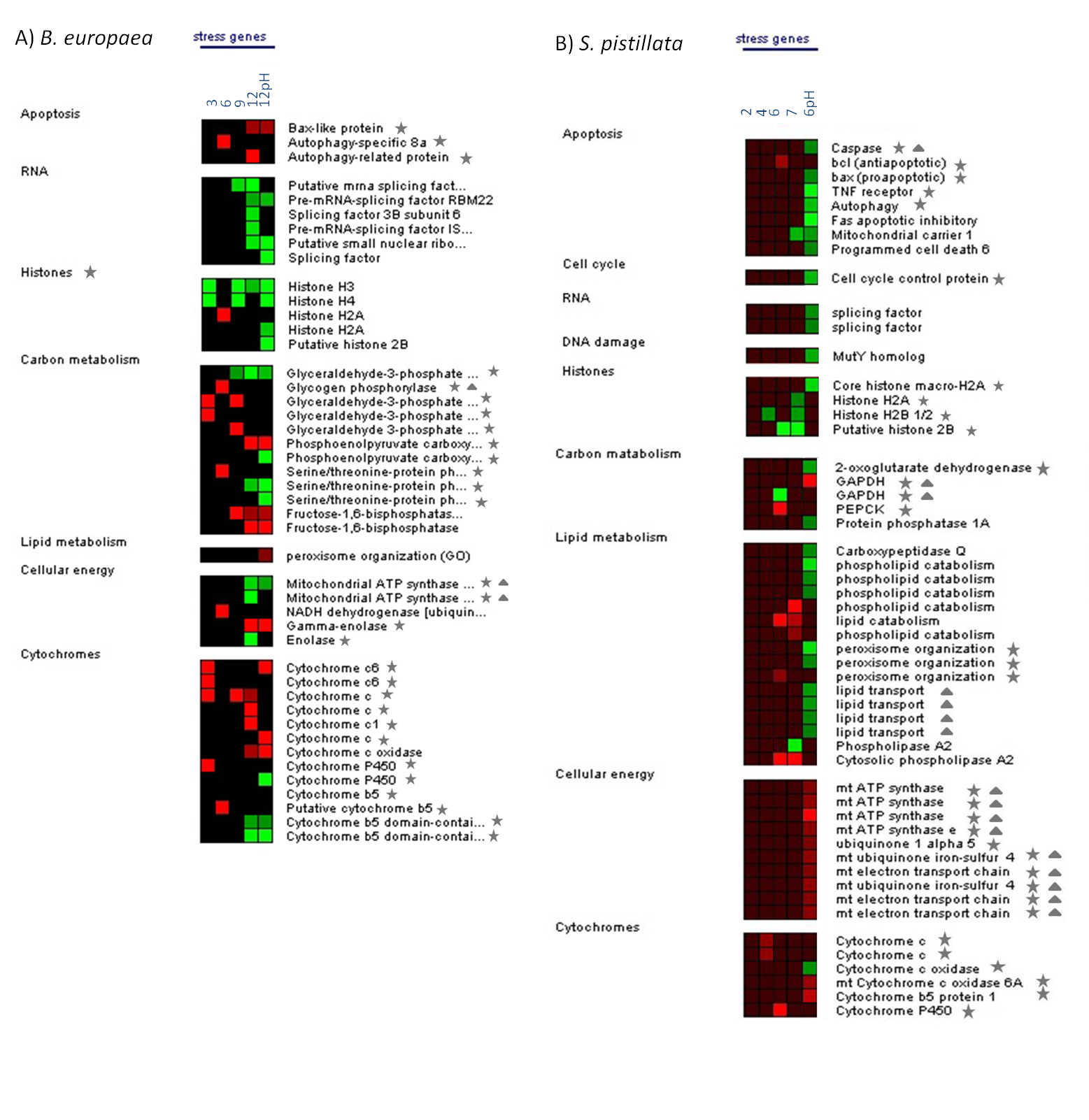


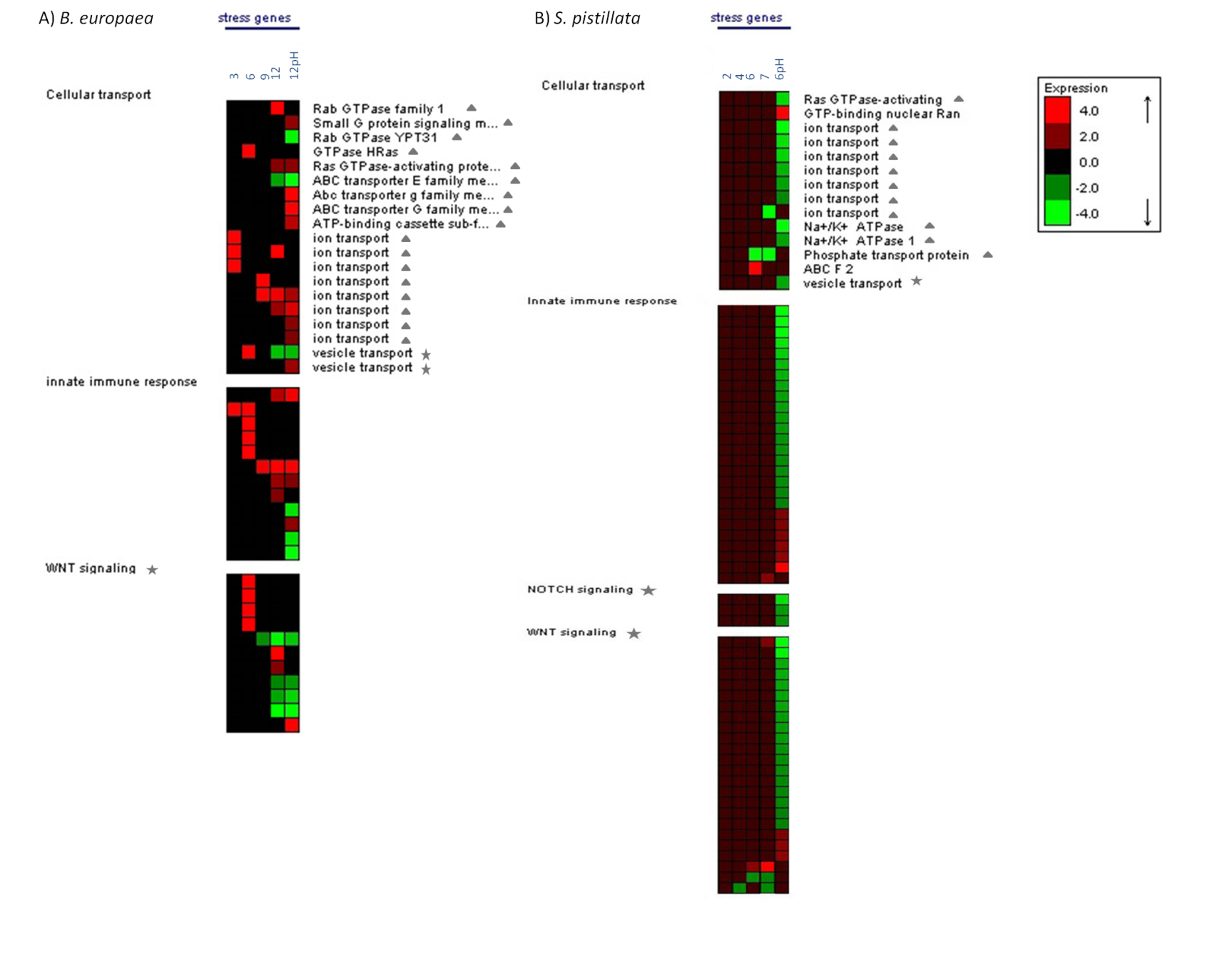


Fig. S4

Heat maps showing fold change values of Cnidarian environmental stress genes in *B. europaea* (A) and *S. pistillata* (B). A list of genes/GOs based on relevant literature was utilized to search the Swissprot and Uniprot50 annotations. The color-scale legend indicating the relative fold change is shown on the right. The environmental stressor is indicated in asterisk – heat and triangle – pH. This figure was constructed using EXPANDER software ^1^. Tables with input data, including contig name, full gene name and Uniprot/Swissprot symbol can be found below (Table S2 and Table S3).


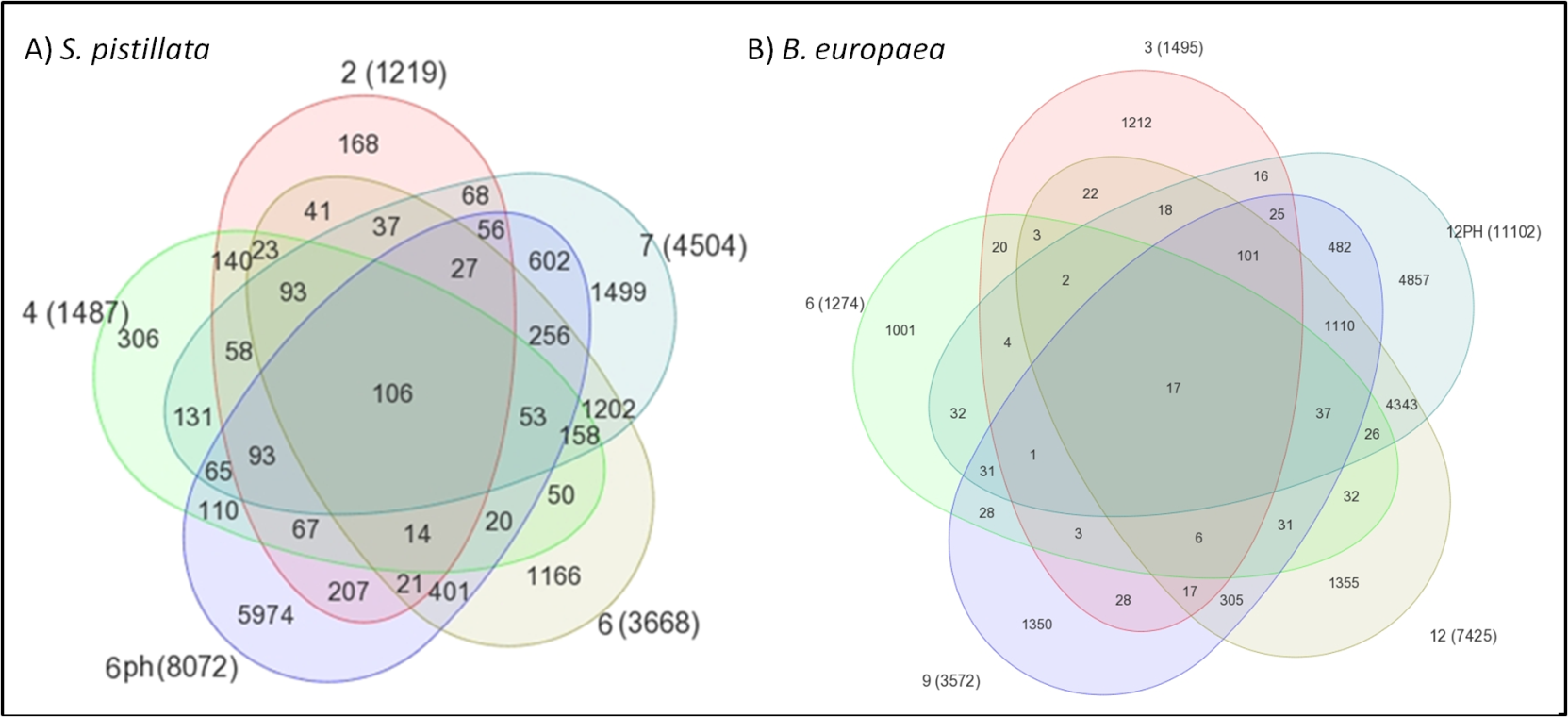


Fig. S5

Venn diagram of all differentially expressed genes in *S. pistillata* (A) and *B. europaea* (B). The number of genes is indicated within each field. 2, 4, 6, 7, 6pH and 3, 6, 9, 12, 12pH, stand for the delta in temperature compared to the control, in *S. pistillata* and *B. europaea*, respectively.


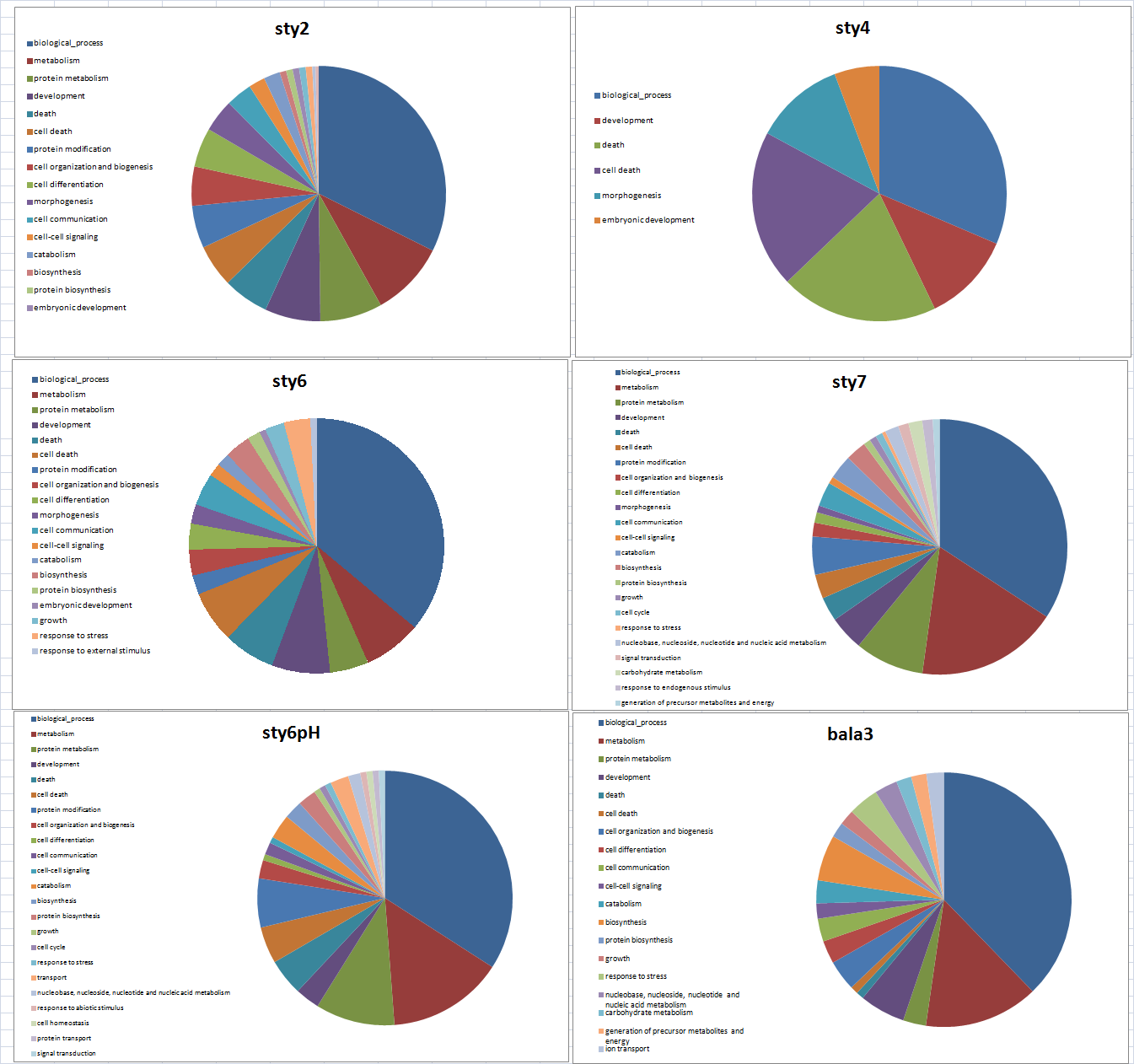
The GO terms assigned to the contig sequences were exported to the web-based CateGOrizer ^2^ in order to generate GO slim terms and to count the ancestors’ terms as a percentage of the total GOs.


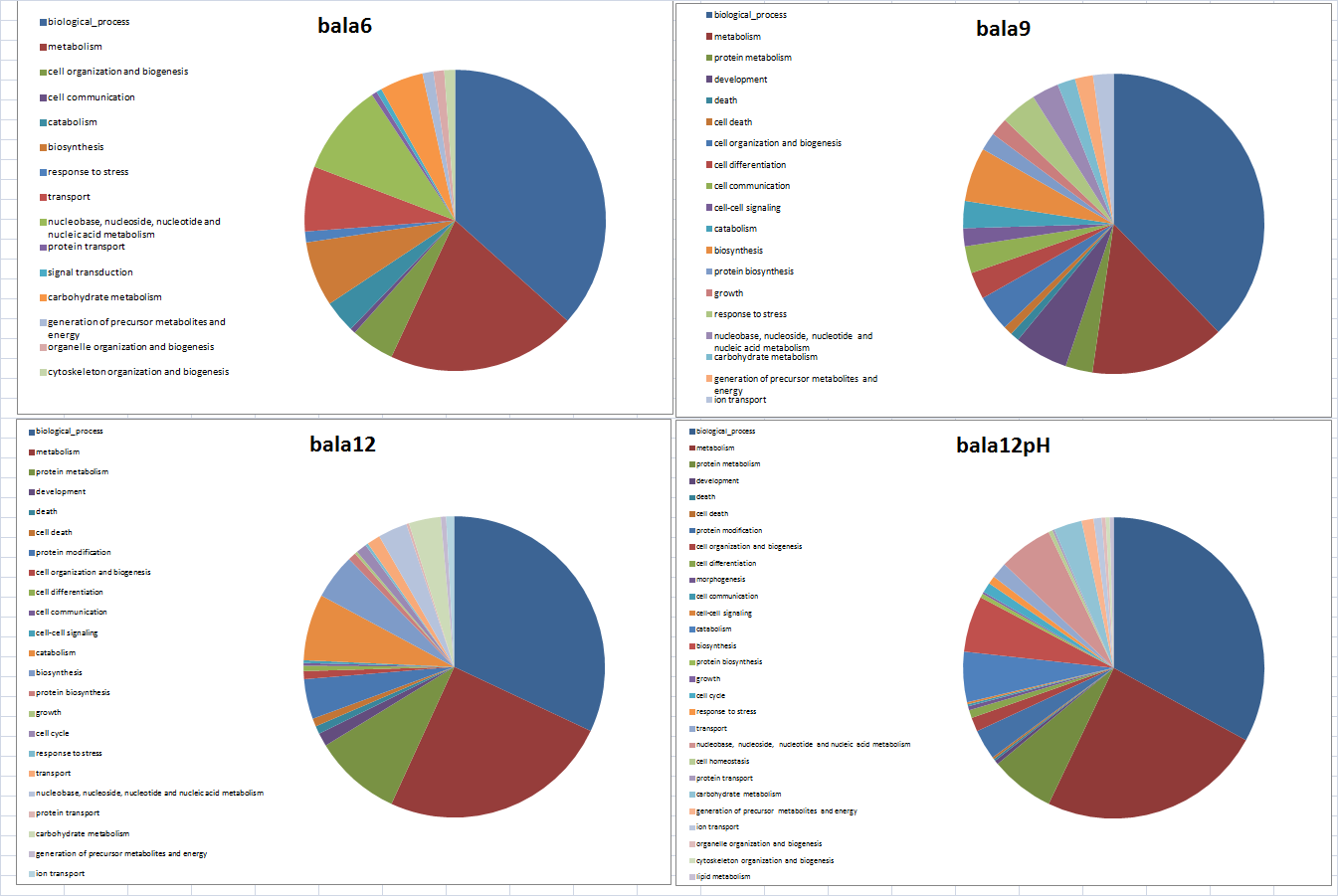


Fig. S6

Pie charts of GO slim terms against coral treatments, based on data from table S6. GO terms were exported to the web-based CateGOrizer^2^ in order to generate GO slim terms and to count the ancestors’ terms as a percentage of the total GOs.


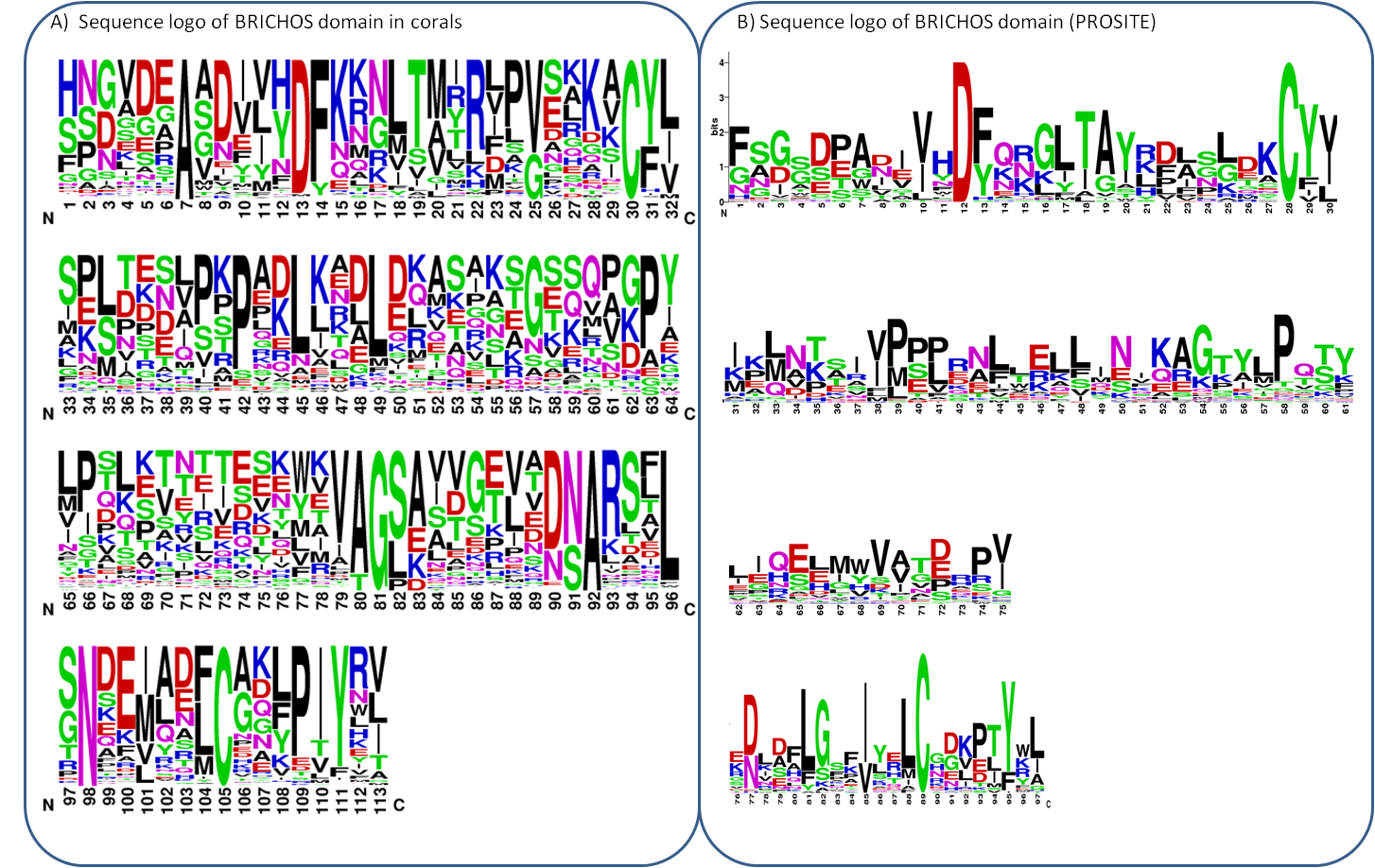


Fig. S7

Sequence logo of BRICHOS domain. A graphical display of a multiple sequence alignment of 15 coral species (A) Compared to the known sequence from PROSITE (B). The sequence logo was generated using WebLogo web tool ^3^.


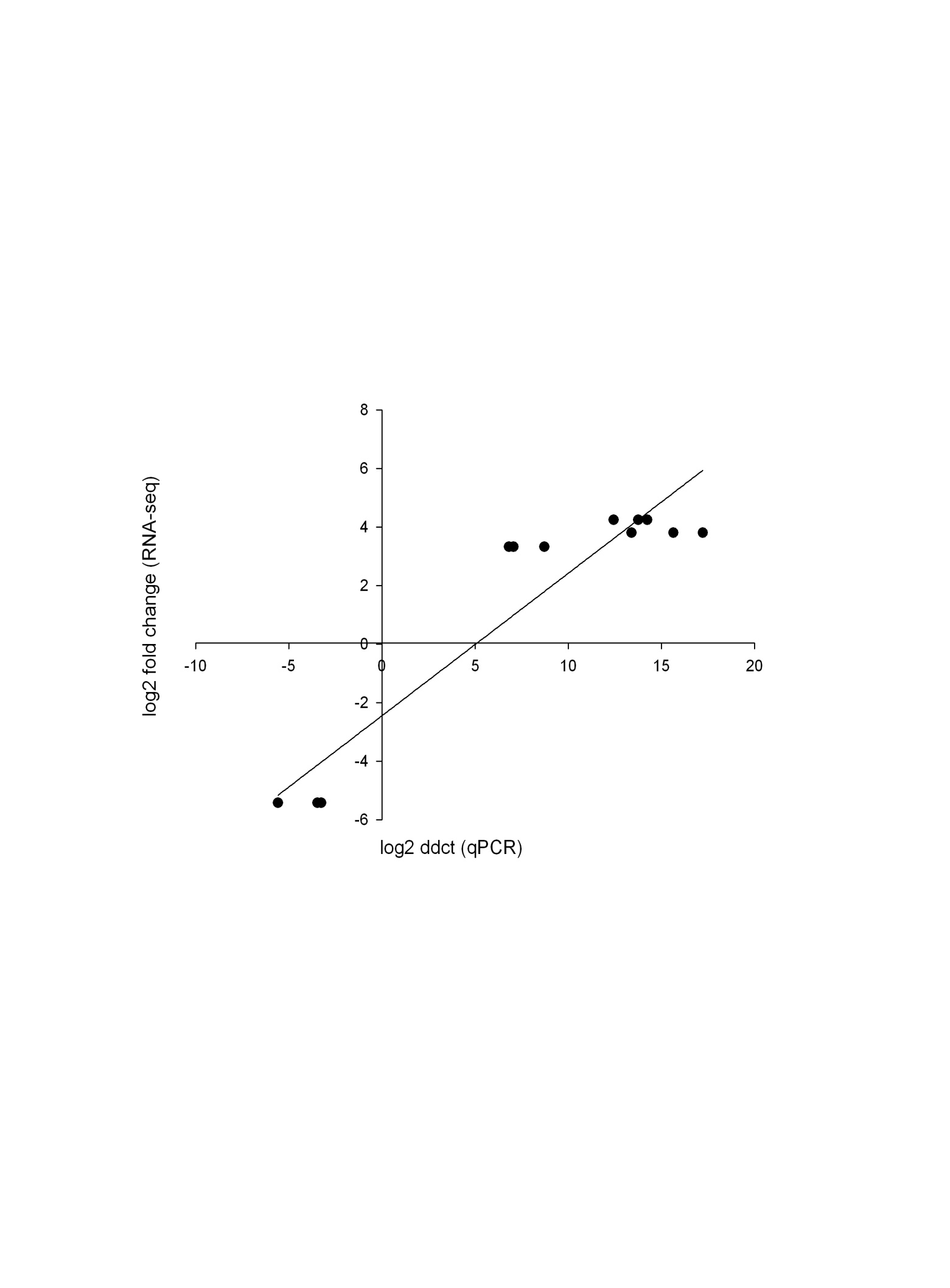


Fig. S8

Correlation of gene expression data between fold-change (RNA-seq) and ddct (quantitative real-time PCR). Log2 fold change in relative gene expression between values obtained from RNA-seq with expression values obtained using qPCR.

Table S1.

Percentage of GO slim terms vs coral treatments. Categories were generated using CateGOrizer ^2^ and based on enriched GOs from David 6.7 (pvalue<0.01). CateGOrizer is a web-based tool utilizing GO slim terms, which are higher level GO categories, and counts the ancestor terms as a percentage of the total GOs.

Table S2.

*B. europaea.* Fold change response by Cnidarian environmental stress genes.

| **Column ID** | **protein name** | **code** | **3** | **6** | **9** | **12** | **12pH** |
| --- | --- | --- | --- | --- | --- | --- | --- |
| **Heat shock proteins** |  |  |  |  |  |  |  |
| comp241028_c0_seq1|m.100364 | HSP | C5LW22 |  |  |  | 7 |  |
| comp487091_c7_seq1|m.819537 | Putative heat shock protein | H2YHC6 |  |  |  | 6 |  |
| comp487463_c0_seq1|m.828121 | HSP90 | P79326 |  | 9 | 12 |  | 18 |
| comp469068_c0_seq1|m.577858 | HSP90 | Q8IC05 |  |  |  |  | 15 |
| comp462931_c0_seq2|m.535854 | HSP90 | Q5QIZ8 |  |  |  |  | 9 |
| comp488044_c1_seq5|m.840911 | 90kDa heat-shock protein | Q9U6R0 |  |  |  |  | 17 |
| comp487091_c5_seq1|m.819525 | HSP90 | I7M3G1 |  |  |  |  | 14 |
| comp478428_c6_seq1|m.664813 | HSP70 | T0XYX6 |  |  |  | 6 |  |
| comp485804_c0_seq8|m.791795 | HSP90 alpha | P07900 |  |  |  |  | 27 |
| comp415731_c0_seq2|m.347177 | Heat shock cognate 90 | B7GEF7 |  |  |  | -4 | -7 |
| comp478428_c11_seq6|m.664844 | Chaperone protein dnaK (HSP70) | Q85FW4 |  |  |  |  | 8 |
| comp485762_c2_seq1|m.791028 | 70 kDa heat shock protein | D0F177 |  |  |  |  | 6 |
| comp478428_c6_seq1|m.664813 | HSP70 | T0XYX6 |  |  |  |  | 5 |
| comp484695_c4_seq6|m.769185 | HSP70 | P11503 |  |  |  |  | 18 |
| comp488105_c4_seq2|m.842170 | Heat shock cognate 71 kDa protein | P11142 |  |  |  |  | 5 |
| comp234834_c0_seq1|m.77704 | HSP70 1a | P08107 |  |  |  | -3 | -4 |
| comp489339_c3_seq3|m.873201 | Heat shock 70 kDa protein 12 | Q39043 |  |  |  |  | 11 |
| comp485762_c3_seq7|m.791059 | Probable mediator of RNA polymerase II transcription subunit 37e (Heat shock 70 kDa protein 1) | P22953 |  |  |  |  | 7 |
| comp485762_c3_seq6|m.791057 | Heat shock cognate protein 70-1 isoform 2 | A0A061ER84 |  |  |  |  | 6 |
| comp443298_c0_seq1|m.436027 | Chaperonin 10 | U3JNU5 |  | 4 |  |  |  |
| comp491682_c0_seq2|m.937175 | Heat shock cognate protein | O22329 |  |  |  |  | -2 |
| comp232753_c0_seq1|m.66427 | Putative DNAJ heat shock protein | F4K9C8 |  |  |  |  | -5 |
| comp467370_c0_seq1|m.565306 | Dnaj heat shock protein family | A0A061RBB2 |  |  |  |  | 6 |
| **oxidative stress** |  |  |  |  |  |  |  |
| comp404159_c0_seq1|m.313903 | Superoxide dismutase | P34697 | 8 |  |  | 7 |  |
| comp403826_c0_seq1|m.313460 | Superoxide dismutase [Mn] | D0N7U0 |  |  | 5 |  | 4 |
| comp483388_c0_seq1|m.745392 | Superoxide dismutase [Mn] | Q9RUV2 |  |  |  |  | 17 |
| comp463875_c0_seq2|m.541411 | Superoxide dismutase [Mn] | Q9PKA0 |  |  |  |  | 17 |
| comp428682_c0_seq1|m.383526 | Putative pln02957 copper zinc superoxide dismutase | G3MH82 |  | 6 |  |  |  |
| comp481218_c0_seq7|m.705583 | Superoxide dismutase [Cu-Zn] | P00445 |  |  | 3 | 3 |  |
| comp425148_c0_seq1|m.372928 | Superoxide dismutase [Cu-Zn] | P34697 |  |  |  |  | 6 |
| comp479888_c0_seq2|m.685253 | Superoxide dismutase [Cu-Zn] | V5G5W5 |  |  |  |  | 4 |
| comp239501_c0_seq2|m.94043 | Putative thioredoxin domain-containing protein 17 | W4XK40 |  | 5 |  |  |  |
| comp456238_c0_seq1|m.497220 | Thioredoxin | I1GF86 |  | 20 |  |  |  |
| comp486827_c4_seq1|m.814341 | Thioredoxin | C5KSW7 |  |  |  | 6 |  |
| comp315454_c0_seq1|m.170371 | Thioredoxin | C5KSW7 |  |  |  | -2 | -3 |
| comp471982_c0_seq2|m.600873 | Thioredoxin-like | C6T0C3 |  |  |  | -4 | -3 |
| comp349329_c0_seq1|m.227309 | Thioredoxin | C5LQN7 |  |  |  | -4 | -4 |
| comp417897_c0_seq1|m.353051 | Thioredoxin | B7G0C9 |  |  |  |  | 6 |
| comp483874_c3_seq2|m.753860 | Thioredoxin | F7GVK7 |  |  |  |  | 3 |
| comp342215_c0_seq1|m.214063 | Thioredoxin | E8Z6X3 |  |  |  |  | -3 |
| comp328572_c0_seq1|m.191575 | Peroxiredoxin-2E | A8HPG8 | 12 |  |  |  |  |
| comp440038_c1_seq1|m.423005 | Peroxiredoxin | B7G6X0 |  |  |  | 6 |  |
| comp242459_c0_seq1|m.105674 | Peroxiredoxin-6 | O77834 |  |  |  | 6 | 6 |
| comp482861_c2_seq3|m.734915 | Peroxiredoxin-1 | P0CB50 |  |  |  |  | 5 |
| comp492818_c0_seq1|m.963690 | Peroxiredoxin-5 | I1SWH6 |  |  |  |  | -4 |
| comp484711_c1_seq1|m.769548 | Catalase-A | O77229 |  |  |  |  | 20 |
| comp484711_c1_seq1|m.769550 | Catalase | A0DRS3 |  |  |  |  | 17 |
| comp473909_c0_seq1|m.617519 | Glutathione S-transferase | J7IB22 |  | 10 |  |  |  |
| comp216057_c0_seq1|m.29434 | Glutathione S-transferase | A0A077JC26 |  |  |  |  | 8 |
| comp500736_c0_seq1|m.1001445 | Glutathione S-transferase | A0A061RJW8 |  |  |  |  | 8 |
| comp435574_c0_seq1|m.404765 | Glutathione S-transferase | A4VDD3 |  |  |  |  | 7 |
| comp239526_c0_seq1|m.94153 | Glutathione S-transferase | Q5YET1 |  |  |  |  | 6 |
| comp470320_c0_seq1|m.587110 | Glutathione S-transferase | B1N8I3 |  |  |  |  | 6 |
| comp234805_c0_seq2|m.77531 | Glutathione S-transferase theta-1 | K1RB85 |  |  |  |  | 3 |
| comp495376_c0_seq1|m.976065 | Glutaredoxin-related protein | A7WQF3 |  |  |  |  | -3 |
| comp407463_c1_seq1|m.322926 | Peroxidase | I4DS54 |  |  |  |  | -4 |
| comp452083_c0_seq1|m.476091 | Ferritin | P42577 | 56 | 12 |  | 10 |  |
| comp470097_c0_seq1|m.585288 | Soma ferritin | A6N9Q6 | 21 |  |  |  | 9 |
| comp473276_c0_seq1|m.611264 | Ferritin | K4HXI8 |  |  |  | 11 |  |
| comp491640_c1_seq1|m.936054 | GFP-like non-fluorescent chromoprotein | P83690 |  |  |  |  | -7 |
| **Nitric-oxide synthesis** |  |  |  |  |  |  |  |
| comp490671_c9_seq3|m.907911 | Nitric oxide synthase, brain | A7SA75 | 4 |  | 12 | 14 | 17 |
| **Protein processing and degradation** |  |  |  |  |  |  |  |
| comp480175_c3_seq1|m.689364 | Calreticulin | E8Z6H5 |  |  | -3 | -3 | -3 |
| comp489263_c5_seq16|m.871155 | Calreticulin | R1D6Y1 |  |  |  |  | 6 |
| comp240736_c0_seq1|m.99067 | Peptidyl-prolyl cis-trans isomerase (PPIase) (EC 5.2.1.8) (Cyclophilin) | Q68PF9 | 10 |  |  |  |  |
| comp506064_c0_seq1|m.1020921 | Peptidyl-prolyl cis-trans isomerase CYP23 (PPIase CYP23) (EC 5.2.1.8) (Cyclophilin of 23 kDa) | A9SEV0 | 5 |  |  |  |  |
| comp495140_c0_seq1|m.974844 | Peptidyl-prolyl cis-trans isomerase CYP18-2 (PPIase CYP18-2) (EC 5.2.1.8) (Cyclophilin of 18 kDa 2) | I3KAH5 |  |  | -2 | -3 | -7 |
| comp494503_c0_seq1|m.971602 | Peptidyl-prolyl cis-trans isomerase CYP22 (PPIase CYP22) (EC 5.2.1.8) (Cyclophilin of 22 kDa) | Q9ZVJ4 |  |  |  | -3 | -4 |
| comp495313_c0_seq1|m.975698 | Peptidyl-prolyl cis-trans isomerase, cyclophilin-type protein | Q23QY9 |  |  |  | -3 | -7 |
| comp503152_c0_seq1|m.1010969 | Peptidyl-prolyl cis-trans isomerase A (PPIase A) (EC 5.2.1.8) (Cyclophilin A) | Q54SM3 |  |  |  |  | 8 |
| comp456974_c1_seq1|m.501097 | Peptidyl-prolyl cis-trans isomerase CYP18-3 (PPIase CYP18-3) (EC 5.2.1.8) (Cyclophilin of 18 kDa 3) | P34790 |  |  |  |  | 6 |
| comp473869_c0_seq4|m.617118 | Peptidyl-prolyl cis-trans isomerase (PPIase) (EC 5.2.1.8) (Cyclophilin) (Cyclosporin A-binding protein) | P21568 |  |  |  |  | -4 |
| comp494746_c0_seq1|m.972881 | Peptidyl-prolyl cis-trans isomerase cyclophilin type | F0SME4 |  |  |  |  | -7 |
| comp487368_c12_seq17|m.825733 | Peptidyl-prolyl cis-trans isomerase B (PPIase B) (EC 5.2.1.8) (Cyclophilin B) (Rotamase B) (S-cyclophilin) | B4KTE5 |  |  |  |  | -23 |
| comp488532_c3_seq10|m.852313 | Calumenin | A7RLD6 |  |  |  | 5 | 5 |
| comp338938_c0_seq1|m.207548 | Prefoldin alpha | L8HDI9 |  |  |  | -6 | -5 |
| comp498894_c0_seq1|m.993816 | Prefoldin subunit | C5L1G3 |  |  |  |  | -3 |
| comp497096_c0_seq1|m.984978 | Prefoldin subunit 4 | C5LZ33 |  |  |  |  | -4 |
| **Ubiquitin** |  |  |  |  |  |  |  |
| comp458706_c0_seq1|m.510570 | Ubiquitin | H8YJF5 |  |  |  | -3 | -4 |
| comp484921_c0_seq4|m.774157 | Ubiquitin | P0DJ25 |  |  |  |  | 6 |
| comp481172_c2_seq1|m.704768 | Ubiquitin | Q5T4S7 |  |  |  |  | 3 |
| comp444557_c1_seq1|m.441365 | Ubiquitin | F0V803 |  |  |  |  | -4 |
| comp348218_c1_seq1|m.225114 | Ubiquitin 1, putative | P35129 |  |  |  |  | -9 |
| comp489157_c0_seq5|m.868331 | Polyubiquitin | K1Q199 |  |  |  |  | 2 |
| comp462277_c0_seq1|m.532182 | Polyubiquitin | A0A076KW18 |  |  |  | 8 |  |
| comp479962_c0_seq5|m.686373 | Polyubiquitin | I7M208 |  |  |  |  | 28 |
| comp475627_c0_seq1|m.634155 | Polyubiquitin | A0A087SDX0 |  |  |  |  | 14 |
| comp338826_c0_seq2|m.207393 | Polyubiquitin-B | M2VTN6 |  |  |  |  | -3 |
| comp427673_c0_seq1|m.380105 | Ubiquitin-conjugating enzyme E2L 3, isoform | P68036 |  |  | 9 |  |  |
| comp475407_c2_seq1|m.631780 | Ubiquitin conjugating enzyme | I7ML74 |  |  |  |  | 6 |
| comp453456_c0_seq10|m.483128 | Putative ubiquitin-conjugating enzyme e2z | Q66KB0 |  |  |  | 3 | 3 |
| comp484815_c7_seq10|m.771998 | Ubiquitin-conjugating enzyme E2W | Q96B02 |  |  |  | 2 | 7 |
| comp232737_c0_seq1|m.66333 | Ubiquitin-conjugating enzyme E2 27 | F0VKF9 |  |  |  | -3 | -3 |
| comp494901_c0_seq1|m.973678 | Ubiquitin-conjugating enzyme E2 N | F0VB00 |  |  |  | -3 | -3 |
| comp494658_c0_seq1|m.972412 | Ubiquitin-conjugating enzyme E2 G1 | Q8I2U3 |  |  |  | -3 | -4 |
| comp232644_c0_seq1|m.65781 | Probable ubiquitin-conjugating enzyme E2 7 | Q4U8F2 |  |  |  | -4 | -3 |
| comp495172_c0_seq1|m.975013 | Ubiquitin-conjugating enzyme E2 | L8GP24 |  |  |  |  | -4 |
| comp497857_c0_seq1|m.988763 | Ubiquitin-conjugating enzyme E2 2 | Q8I2Q2 |  |  |  |  | -3 |
| comp455951_c0_seq4|m.495770 | Ubiquitin-conjugating enzyme E2 | A0A074TPQ5 |  |  |  |  | -3 |
| comp233094_c0_seq1|m.68437 | Probable ubiquitin-conjugating enzyme E2 7 | P0CG47 |  |  |  |  | -3 |
| comp496250_c0_seq1|m.980725 | NEDD8-conjugating enzyme Ubc12 | C5LTL4 |  |  |  |  | -7 |
| comp354826_c0_seq1|m.237744 | Ubiquitin-conjugating enzyme E2 W | P0CH08 |  |  |  |  | 7 |
| comp402469_c0_seq1|m.311390 | Ubiquitin-conjugating enzyme E2 W | L8H1Q6 |  |  |  |  | 4 |
| comp489829_c11_seq2|m.885223 | Ubiquitin-conjugating enzyme E2 S | Q14139 |  |  |  |  | 2 |
| comp485457_c1_seq2|m.785154 | Ubiquitin-conjugating enzyme E2 Z | T2MCP0 |  |  |  |  | 2 |
| comp469582_c0_seq1|m.581770 | Putative scf ubiquitin ligase skp1 component | P63208-2 |  |  | 7 |  |  |
| comp466554_c1_seq3|m.559104 | E3 ubiquitin-protein ligase | Q15907 |  |  | 17 |  |  |
| comp465811_c0_seq1|m.553841 | ubiquitin ligase complex protein SKP1a | D0MV20 |  |  |  | 6 |  |
| comp472117_c2_seq4|m.602050 | Putative e3 ubiquitin ligase | Q5XHH7 |  |  |  | 3 | 3 |
| comp475027_c2_seq3|m.628249 | Putative E3 ubiquitin-protein ligase | P49632 |  |  |  |  | 11 |
| comp488554_c8_seq1|m.852805 | putative ubiquitin-protein ligase | Q9VX25 |  |  |  | 2 | 2 |
| comp338999_c0_seq1|m.207719 | ubiquitin-protein ligase | Q5T447 |  |  |  | 2 |  |
| comp482506_c2_seq10|m.729205 | E3 ubiquitin-protein ligase | Q14669 |  |  |  | 2 | 3 |
| comp465811_c0_seq15|m.553844 | ubiquitin ligase complex protein SKP1a | D0MV20 |  |  |  | -2 | -5 |
| comp482214_c0_seq1|m.723003 | E3 ubiquitin-protein ligase synoviolin B | H0Y659 |  |  |  |  | 3 |
| comp495837_c0_seq1|m.978546 | ubiquitin ligase complex protein | D8SNW8 |  |  |  |  | -6 |
| comp485467_c0_seq14|m.785386 | Putative E3 ubiquitin-protein ligase | Q9UKV5 |  |  |  |  | 2 |
| comp349878_c0_seq1|m.228383 | Ubiquitin carboxyl-terminal hydrolase | P62068 |  |  |  |  | 2 |
| comp484921_c0_seq1|m.774152 | Ubiquitin carboxyl-terminal hydrolase | B0B030 |  |  |  |  | 3 |
| comp471147_c0_seq1|m.593671 | Ubiquitin carboxyl-terminal hydrolase | A7SHS4 |  |  |  |  | 2 |
| comp489326_c0_seq5|m.872894 | Ubiquitin carboxyl-terminal hydrolase | P62972 |  |  |  |  | 2 |
| comp500747_c0_seq1|m.1001484 | Small ubiquitin-related modifier 1 | Q6DEP7 |  |  |  | 8 | 8 |
| comp231375_c0_seq1|m.59795 | Small ubiquitin-related modifier 2 | G5BNC2 |  |  |  | 7 | 8 |
| comp494111_c0_seq1|m.969658 | Small ubiquitin-related modifier 2-B | C5KAH8 |  |  |  | -4 |  |
| comp215891_c0_seq1|m.29123 | Small ubiquitin-related modifier 1 | P46595 |  |  |  |  | 8 |
| comp455152_c0_seq1|m.491433 | Ubiquitin-40S ribosomal protein S27a | A0A087RB55 |  |  |  | 25 |  |
| comp489326_c0_seq5|m.872894 | Putative ubiquitin a-52 residue ribosomal protein fusion product 1 | P62972 |  |  |  | 3 | 2 |
| comp407346_c0_seq1|m.322589 | Ubiquitin-60S ribosomal protein | M2VTN6 |  |  |  |  | 7 |
| comp372587_c1_seq1|m.268168 | Ubiquitin-60S ribosomal protein L40 | A0A078ABQ1 |  |  |  |  | 6 |
| comp475407_c2_seq1|m.631777 | Ubiquitin-activating enzyme E1 | A0A077ZY60 |  |  |  |  | 5 |
| comp482598_c9_seq5|m.730636 | Ubiquitin-activating enzyme e1 | Q99496 |  |  |  |  | 4 |
| comp479962_c0_seq11|m.686384 | Ubiquitin-related protein 2 | I2H6L2 |  |  |  | 2 |  |
| comp346936_c1_seq1|m.222818 | Proteasomal ubiquitin receptor ADRM1 | A7S704 |  |  |  |  | 2 |
| comp464636_c2_seq1|m.546585 | Proteasomal ubiquitin receptor ADRM1 | A7S8P2 |  |  |  |  | 2 |
| **apoptosis** |  |  |  |  |  |  |  |
| comp480658_c1_seq1|m.696530 | Bax-like protein | B7SB94 |  |  |  | 2 | 3 |
| comp488353_c2_seq7|m.848508 | Autophagy-specific 8a-like protein | Q28FC7 |  | 23 |  |  |  |
| comp259977_c0_seq1|m.122413 | Autophagy-related protein | Q8H715 |  |  |  | 5 |  |
| **cytoskeleton and extracellular skeleton** |  |  |  |  |  |  |  |
| comp487437_c1_seq9|m.827553 | Actin | A0AUL6 | 3 | 3 | 7 | 5 |  |
| comp472980_c0_seq7|m.609007 | Actin | Q766D6 |  |  | 5 |  |  |
| comp488332_c5_seq1|m.847773 | Actin | Q8BF62 |  |  |  | 43 |  |
| comp488332_c5_seq1|m.847773 | Actin | Q8BF62 |  |  |  |  | 42 |
| comp472980_c0_seq7|m.609007 | Actin | Q766D6 |  |  |  |  | 9 |
| comp459872_c1_seq1|m.517899 | Actin | P24902 |  |  |  |  | 5 |
| comp491541_c1_seq24|m.933334 | Actin-1 | P10982 |  |  |  |  | -13 |
| comp491541_c1_seq6|m.933301 | Actin 5C | C7TYF9 |  |  |  |  | -9 |
| comp488332_c0_seq2|m.847760 | Actin 3 | O61377 |  |  |  |  | 12 |
| comp477882_c0_seq1|m.658609 | Actin-related protein 2/3 complex subunit 3 | O15145 |  | 12 |  |  |  |
| comp466218_c0_seq1|m.556970 | Putative f-actin capping protein beta subunit | P47757 |  | 10 |  |  |  |
| comp465445_c0_seq1|m.551441 | Actin-related protein 2/3 complex subunit 5 | A3QX08 |  | 12 |  |  |  |
| comp421004_c0_seq1|m.361247 | F-actin capping protein alpha subunit-like protein | Q9W2N0 |  | 16 |  |  |  |
| comp445148_c0_seq1|m.443696 | Actin depolyrising factor 2 | M9PA58 |  |  |  |  | 15 |
| comp506901_c0_seq1|m.1023021 | Beta-tubulin folding cofactor A | H3B7B0 |  | 6 |  |  |  |
| comp490831_c4_seq1|m.912012 | Tubulin beta chain | P02556 |  |  |  |  | 32 |
| comp479378_c0_seq2|m.678921 | Tubulin alpha-3 chain | X6NUE3 |  |  | 11 | 3 | 8 |
| comp490028_c2_seq1|m.890264 | Tubulin gamma chain | P83887 |  |  |  | -4 | -4 |
| comp497076_c0_seq1|m.984877 | Tubulin folding cofactor B | A8I1X1 |  |  |  | -4 | -4 |
| comp482631_c0_seq1|m.731146 | Cysteine-rich protein | A4QNF7 |  | 9 |  |  |  |
| comp487416_c0_seq31|m.827130 | Lectin | D4QD85 | 3 |  | 3 |  | 6 |
| comp233167_c0_seq2|m.68730 | Mannose-binding lectin associated serine protease | B9X085 |  |  |  |  | 3 |
| comp490168_c0_seq22|m.893725 | Collagen triple helix repeat-containing protein 1 | A7S663 | 3 |  | 4 | 12 | 6 |
| comp473615_c0_seq13|m.614621 | Cuticle collagen 14 | A0A077Z745 |  |  |  | 7 |  |
| comp473615_c0_seq13|m.614621 | Cuticle collagen 14 | A0A077Z745 |  |  |  |  | 3 |
| comp477354_c0_seq1|m.652750 | Collagen alpha-1(IV) chain | V9GW22 |  |  |  | 3 |  |
| comp480162_c7_seq6|m.689143 | Prolyl 4-hydroxylase subunit alpha-1 (4-PH alpha-1) | A7SJ24 |  |  |  | 2 | 3 |
| comp479387_c4_seq1|m.679036 | Peroxidasin-like protein | V9GVG1 |  |  |  | 3 |  |
| comp486263_c0_seq1|m.802324 | Protocadherin-like protein | B8V7Q1 |  |  |  |  | 2 |
| comp489957_c0_seq81|m.888749 | Carbonic anhydrase | Q5ENU2 |  |  |  |  | -8 |
| comp503120_c0_seq1|m.1010859 | Profilin | Q8GSL5 | 9 |  |  |  |  |
| comp437967_c0_seq1|m.414486 | Dynein light chain 2B | H2XQ56 |  | 5 |  |  |  |
| comp495098_c0_seq1|m.974665 | Putative dynein light chain roadblock-type 2 | S8GCV3 |  | -3 |  |  | -7 |
| comp486372_c0_seq3|m.804831 | Myosin regulatory light chain | P80164 |  | 9 | 14 |  |  |
| comp478505_c0_seq1|m.665857 | Tropomyosin | A5D6I4 |  |  | 6 |  | 13 |
| comp468427_c0_seq1|m.572894 | Myosin light chain | E7CGC3 |  |  | 6 |  | 21 |
| comp482187_c0_seq2|m.722671 | Essential myosin light chain | U1NYW0 |  |  |  | 6 |  |
| comp492252_c2_seq1|m.954716 | Myosin heavy chain | G4M0G3 |  |  |  |  | 9 |
| comp492252_c2_seq2|m.954735 | Myosin heavy chain | O96063 |  |  |  |  | 9 |
| comp491834_c3_seq4|m.941952 | Myosin heavy chain, muscle | P05661 |  |  |  |  | 6 |
| comp490576_c0_seq4|m.905135 | Myosin regulatory light chain 1 | P19625 |  |  |  |  | 6 |
| comp232617_c0_seq1|m.65645 | Myosin II essential light chain | A7RMQ1 |  |  |  |  | -3 |
| comp233219_c0_seq1|m.69015 | Myosin regulatory light chain | P40423 |  |  |  |  | -6 |
| comp492578_c0_seq1|m.962785 | Predicted protein Tropomyosin | Q6S5G5 |  |  |  |  | -6 |
| comp492603_c0_seq1|m.962851 | Myosin II essential light chain | A7RMQ2 |  |  |  |  | -7 |
| **calcium homeostasis** |  |  |  |  |  |  |  |
| comp483072_c0_seq1|m.738554 | Calcium/calmodulin-dependent protein kinase type II | O62305-4 |  | 5 |  |  |  |
| comp467848_c0_seq1|m.568776 | Putative calmodulin | P29289 |  |  | 3 |  |  |
| comp484404_c0_seq4|m.764093 | Calmodulin | P27165 |  |  | -2 |  |  |
| comp485480_c0_seq3|m.785546 | Calmodulin | P27165 |  |  |  | 9 | 6 |
| comp484404_c0_seq3|m.764088 | Calmodulin | P27165 |  |  |  | -3 | -4 |
| comp363362_c0_seq1|m.255063 | Calmodulin | P07463 |  |  |  |  | -2 |
| comp235584_c0_seq1|m.81983 | Calmodulin | C5KAW4 |  |  |  |  | -3 |
| comp498539_c0_seq1|m.992129 | Calmodulin | P02599 |  |  |  |  | -4 |
| comp496845_c0_seq1|m.983697 | Calmodulin | C5LTF4 |  |  |  |  | -4 |
| comp487370_c11_seq1|m.825833 | Calcium-transporting ATPase | A7S3H9 |  |  |  | 2 |  |
| comp491295_c0_seq7|m.925852 | Plasma membrane calcium-transporting ATPase 3 | Q6UUX1 |  |  |  |  | 2 |
| **carbon metabolism** |  |  |  |  |  |  |  |
| comp489249_c0_seq24|m.870812 | Glyceraldehyde-3-phosphate dehydrogenase | Q27890 |  |  | -2 | -4 | -3 |
| comp488470_c0_seq2|m.851078 | Glycogen phosphorylase | P11216 |  | 4 |  |  |  |
| comp489612_c0_seq6|m.879915 | Glyceraldehyde-3-phosphate dehydrogenase | P25857 | 12 |  | 7 |  |  |
| comp489612_c0_seq21|m.879955 | Glyceraldehyde-3-phosphate dehydrogenase | E3SCB8 | 9 |  |  |  |  |
| comp471439_c2_seq1|m.596331 | Glyceraldehyde 3-phosphate dehydrogenase domain-containing protein 4_6 | Q4JHJ4 |  |  | 6 |  |  |
| comp457160_c0_seq5|m.501907 | Phosphoenolpyruvate carboxykinase | Q2S008 |  |  |  | 5 | 6 |
| comp485961_c0_seq10|m.795183 | Phosphoenolpyruvate carboxylase | B9X0T7 |  |  |  |  | -6 |
| comp467063_c0_seq3|m.563323 | Serine/threonine-protein phosphatase PP1-beta | P36873 |  | 5 |  |  |  |
| comp494295_c0_seq1|m.970566 | Serine/threonine-protein phosphatase PP1 | Q9UW86 |  |  |  | -3 | -5 |
| comp497034_c0_seq1|m.984653 | Serine/threonine-protein phosphatase PP1 | P48489 |  |  |  |  | -4 |
| comp483479_c0_seq1|m.746970 | Fructose-1,6-bisphosphatase isozyme 2 | Q9Z1N1 |  |  | 7 | 3 | 3 |
| comp501009_c0_seq1|m.1002505 | Fructose-1,6-bisphosphatase | A3QST0 |  |  |  | 6 | 5 |
| **lipid matabolism** |  |  |  |  |  |  |  |
| comp475180_c0_seq3|m.629564 | peroxisome organization (GO) | Q86WA8 |  |  |  |  | 2 |
| **cellular transport** |  |  |  |  |  |  |  |
| comp240646_c0_seq1|m.98703 | Rab GTPase family 1 | Q01890 |  |  |  | 5 |  |
| comp479821_c1_seq7|m.684255 | Small G protein signaling modulator 3 | Q96HU1 |  |  |  |  | 2 |
| comp235121_c0_seq1|m.79386 | Rab GTPase YPT31 | P38555 |  |  |  |  | -4 |
| comp468970_c4_seq1|m.577038 | GTPase HRas | B3S5R4 |  | 15 |  |  |  |
| comp484039_c8_seq38|m.756681 | Ras GTPase-activating protein 1 | A7SJB4 |  |  |  | 2 | 2 |
| comp236318_c0_seq1|m.84487 | ABC transporter E family member 2 | G7IE72 |  |  |  | -3 | -5 |
| comp483662_c0_seq1|m.749931 | Abc transporter g family member 22-like | A0A061S2M1 |  |  |  |  | 9 |
| comp463796_c0_seq1|m.540833 | ABC transporter G family member 7 | A8IYC3 |  |  |  |  | 5 |
| comp487743_c6_seq14|m.833478 | ATP-binding cassette sub-family B member 7, mitochondrial | O75027 |  |  |  |  | 3 |
| comp413950_c0_seq1|m.342801 | ion transport (GO) | Q84ZX2 | 23 |  |  |  |  |
| comp319995_c0_seq2|m.177183 | ion transport (GO) | A4RX32 | 6 |  |  | 5 |  |
| comp503599_c0_seq1|m.1012814 | ion transport (GO) | Q5S7Y5 | 5 |  |  |  |  |
| comp475441_c0_seq4|m.632116 | ion transport (GO) | A8HXJ4 |  |  | 6 |  |  |
| comp489157_c0_seq9|m.868339 | ion transport (GO) | C3XUD0 |  |  | 4 | 4 | 3 |
| comp483861_c1_seq1|m.753478 | ion transport (GO) | A7RN63 |  |  |  | 2 | 3 |
| comp476184_c2_seq9|m.639677 | ion transport (GO) | Q9UJZ1 |  |  |  |  | 2 |
| comp486367_c4_seq15|m.804706 | ion transport (GO) | O00141 |  |  |  |  | 2 |
| comp475498_c0_seq2|m.632684 | vesicle transport (Go) | P62820 |  | 13 |  | -3 | -3 |
| comp458867_c0_seq1|m.511620 | vesicle transport (Go) | Q02248 |  |  |  |  | 2 |
| **RNA** |  |  |  |  |  |  |  |
| comp496932_c0_seq1|m.984148 | Putative mrna splicing factor atp-dependent rna helicase | Q9VIZ3 |  |  | -3 | -4 |  |
| comp495868_c0_seq1|m.978659 | Pre-mRNA-splicing factor RBM22 | C5LG19 |  |  |  | -3 | -3 |
| comp493690_c0_seq1|m.967593 | Splicing factor 3B subunit 6 | Q7XZG6 |  |  |  | -4 |  |
| comp412162_c0_seq1|m.337175 | Pre-mRNA-splicing factor ISY1-like protein | Q9VHV6 |  |  |  | -4 |  |
| comp288114_c0_seq1|m.141849 | Putative small nuclear ribonucleoprotein snrnp splicing factor | B7PH70 |  |  |  | -4 | -5 |
| comp494796_c0_seq1|m.973149 | Splicing factor | C5LN30 |  |  |  |  | -5 |
| **cellular energy** |  |  |  |  |  |  |  |
| comp493913_c0_seq1|m.968669 | Mitochondrial ATP synthase F1 epsilon subunit-like protein 1 | F2WQ50 |  |  |  | -3 | -3 |
| comp232428_c0_seq1|m.64612 | Mitochondrial ATP synthase oligomycin sensitivity-conferring protein | F2WQ48 |  |  |  | -5 |  |
| comp402433_c0_seq1|m.311332 | NADH dehydrogenase [ubiquinone] 1 alpha subcomplex subunit 2 | E9CGX9 |  | 4 |  |  |  |
| comp437758_c2_seq5|m.413438 | Gamma-enolase | A6QR19 |  |  |  | 6 | 4 |
| comp232471_c0_seq1|m.64837 | Enolase | P25696 |  |  |  | -4 |  |
| **cytochromes** |  |  |  |  |  |  |  |
| comp493982_c0_seq1|m.969091 | Cytochrome c6 | Q8GTB4 | 50 |  |  |  | 13 |
| comp329843_c0_seq1|m.193447 | Cytochrome c6 | A0A061R2G0 | 9 |  |  |  |  |
| comp419894_c0_seq1|m.358135 | Cytochrome c | A0A061RYV8 | 8 |  | 5 | 3 |  |
| comp445767_c0_seq1|m.446295 | Cytochrome c | P99999 |  |  |  | 10 |  |
| comp398604_c0_seq1|m.305414 | Cytochrome c1 | B8LDW8 |  |  |  | 5 |  |
| comp493853_c0_seq1|m.968374 | Cytochrome c | P00079 |  |  |  |  | 14 |
| comp484915_c2_seq24|m.773971 | Cytochrome c oxidase | A7RII8 |  |  |  | 3 | 5 |
| comp449768_c0_seq1|m.464020 | Cytochrome P450 | W5H1Y8 | 8 |  |  |  |  |
| comp503223_c0_seq1|m.1011207 | Cytochrome P450 | UPI0001CBB858 |  |  |  |  | -4 |
| comp486854_c0_seq3|m.814971 | Cytochrome b5 | H2ZAX0 |  |  |  |  |  |
| comp453039_c0_seq1|m.481005 | Putative cytochrome b5 | C5LX93 |  | 5 |  |  |  |
| comp471659_c0_seq1|m.598073 | Cytochrome b5 domain-containing protein | A0A074T5N9 |  |  |  | -3 | -2 |
| comp495176_c0_seq1|m.975027 | Cytochrome b5 domain-containing protein 1 | A0A074T5N9 |  |  |  | -4 | -5 |
| **Histones** |  |  |  |  |  |  |  |
| comp487661_c4_seq24|m.831776 | Histone H3 | Q71DI3 | -4 |  | -4 | -3 | -4 |
| comp478691_c0_seq1|m.668761 | Histone H4 | H2QSG4 | -5 |  | -5 |  | -5 |
| comp429096_c0_seq1|m.384602 | Histone H2A | P08985 |  | 10 |  |  |  |
| comp486009_c4_seq5|m.796258 | Histone H2A | I3KZV4 |  |  |  |  | -3 |
| comp492948_c0_seq1|m.964216 | Putative histone 2B | E3UGH3 |  |  |  |  | -6 |
| **innate immune response (GO)** |  |  |  |  |  |  |  |
| comp481825_c1_seq6|m.715831 | innate immune response (GO) | A0T397 |  |  |  | 3 | 4 |
| comp242478_c0_seq1|m.105725 | innate immune response (GO) | P07858 | 6 | 6 |  |  |  |
| comp465004_c0_seq1|m.548671 | innate immune response (GO) | P63098 |  | 6 |  |  |  |
| comp428050_c0_seq1|m.381464 | innate immune response (GO) | P61088 |  | 5 |  |  |  |
| comp458552_c0_seq1|m.509602 | innate immune response (GO) | P62993 |  | 7 |  |  |  |
| comp488667_c2_seq9|m.855758 | innate immune response (GO) | A7SLZ2 |  |  | 4 | 4 | 4 |
| comp490210_c0_seq1|m.894876 | innate immune response (GO) | P12931 |  |  |  | 2 | 2 |
| comp418430_c0_seq1|m.354416 | innate immune response (GO) | P41240 |  |  |  | 2 |  |
| comp495964_c0_seq1|m.979160 | innate immune response (GO) | Q9SZD4 |  |  |  | -4 |  |
| comp490445_c0_seq20|m.901331 | innate immune response (GO) | P15336 |  |  |  |  | 2 |
| comp495964_c0_seq1|m.979160 | innate immune response (GO) | Q9SZD4 |  |  |  |  | -4 |
| comp232604_c0_seq1|m.65572 | innate immune response (GO) | Q13200 |  |  |  |  | -6 |
| **WNT signaling** |  |  |  |  |  |  |  |
| comp394575_c0_seq1|m.299080 | WNT signaling (GO) | P25787 |  | 6 |  |  |  |
| comp461909_c0_seq1|m.529904 | WNT signaling (GO) | P48148 |  | 18 |  |  |  |
| comp487850_c0_seq2|m.836931 | WNT signaling (GO) | P30153 |  | 4 |  |  |  |
| comp478153_c0_seq1|m.661366 | WNT signaling (GO) | P68400 |  | 6 |  |  |  |
| comp494887_c0_seq1|m.973608 | WNT signaling (GO) | P60900 |  |  | -2 | -4 | -3 |
| comp242379_c0_seq1|m.105401 | WNT signaling (GO) | P63244 |  |  |  | 5 |  |
| comp435302_c0_seq5|m.403563 | WNT signaling (GO) | P22727 |  |  |  | 2 |  |
| comp257769_c0_seq1|m.120309 | WNT signaling (GO) | P62333 |  |  |  | -2 | -2 |
| comp445302_c3_seq1|m.444308 | WNT signaling (GO) | A7RF25 |  |  |  | -3 | -3 |
| comp344061_c0_seq1|m.217674 | WNT signaling (GO) | P48729 |  |  |  | -5 | -5 |
| comp472467_c0_seq2|m.604713 | WNT signaling (GO) | P67775 |  |  |  |  | 10 |

Table S3.

*S. pistillata.* Fold change response of Cnidarian environmental stress genes.

| **Column ID** | **protein name** | **code** | **6pH** | **7** | **6** | **4** | **2** |
| --- | --- | --- | --- | --- | --- | --- | --- |
| **Heat shock proteins** |  |  |  |  |  |  |  |
| comp178787_c1_seq5|m.399593 | small heat shock protein | Q0H8V6 | 8 | 44 | 19 |  |  |
| comp169378_c1_seq2|m.224616 | Protein associated with small stress protein 1 | A7SQQ9 |  |  | 4 |  |  |
| comp176787_c0_seq1|m.351272 | GRP-78 | P11021 | 3 | 3 | 3 |  |  |
| comp175044_c0_seq1|m.316489 | Endoplasmin (Heat shock protein 90 kDa beta member 1) | Q66HD0 | 3 | 3 |  |  |  |
| comp167356_c1_seq8|m.202112 | Heat shock 70 | A7RF55 | 2 |  |  |  |  |
| comp178293_c1_seq1|m.387868 | Heat shock 70 | P38646 |  | 2 |  |  |  |
| comp160147_c1_seq2|m.155056 | HSP 90-alpha | P07900 |  | 2 | 2 |  |  |
| comp180584_c0_seq4|m.445492 | Heat shock protein 86 | Q8IC05 | 5 |  |  |  |  |
| comp181706_c1_seq1|m.477434 | DnaJ homolog subfamily A member 1 | P31689 |  |  | 2 |  |  |
| comp163546_c0_seq2|m.170795 | DnaJ homolog subfamily C member 3 | Q13217 |  | 2 |  |  |  |
| **Oxidative stress** |  |  |  |  |  |  |  |
| comp175823_c2_seq2|m.331108 | Thioredoxin-like protein 1 | O43396 | 2 |  |  |  |  |
| comp144547_c0_seq3|m.119671 | Thioredoxin domain-containing protein 9 | O64628 | 6 |  |  |  | 3 |
| comp170686_c2_seq10|m.241712 | Peroxiredoxin-6 | P30041 | 3 | 2 | 2 |  |  |
| comp159535_c0_seq1|m.152899 | Peroxiredoxin-6 | Q5ZJF4 | 2 |  | 2 |  |  |
| comp182825_c0_seq1|m.503697 | Peroxiredoxin-5 | I1SWH6 | 4 |  |  |  |  |
| comp171895_c0_seq1|m.261022 | Catalase | P04040 |  |  | 3 |  |  |
| comp182740_c0_seq1|m.503474 | glutathione S-transferase 3 | A3E3T8 | 6 |  |  |  |  |
| comp179486_c0_seq1|m.416677 | Hypoxia inducible factor | A0A076FS22 |  |  | 2 |  |  |
| comp171831_c1_seq18|m.259815 | Green fluorescent protein | B0ZZ77 | 4 | 7 | 2 | 3 | 4 |
| **Protein processing and degradation** |  |  |  |  |  |  |  |
| Ubiquitin |  |  |  |  |  |  |  |
| comp173872_c0_seq1|m.293367 | E3 ubiquitin-protein ligase | P61092 | -4 |  |  |  |  |
| comp163855_c0_seq1|m.172696 | E3 ubiquitin-protein ligase | Q8WY64 | -4 | -2 | -3 |  |  |
| comp179288_c1_seq1|m.412266 | Ubiquitin ligase | E0VYN4 | -3 |  |  |  |  |
| comp174788_c3_seq2|m.311668 | E3 ubiquitin-protein ligase | T2M440 | -3 |  |  |  |  |
| comp160020_c1_seq1|m.154564 | E3 ubiquitin-protein ligase | Q9H4P4 | -3 |  |  |  |  |
| comp177266_c1_seq5|m.362475 | E3 ubiquitin-protein ligase | Q6Q0C0 | -2 |  |  |  |  |
| comp159670_c1_seq2|m.153440 | Ubiquitin-like protein 3 | O95164 | -3 |  |  |  |  |
| comp176623_c0_seq2|m.348007 | Ubiquitin thioesterase | Q9UGI0 | -3 |  |  |  |  |
| comp155369_c3_seq1|m.140682 | G2/M phase-specific E3 ubiquitin-protein ligase | K1QSM3 | -3 |  |  |  |  |
| comp175343_c3_seq2|m.322289 | Ubiquitin-conjugating enzyme E2 | P61086 | -2 |  |  |  |  |
| comp180445_c5_seq1|m.441520 | Ubiquitin-conjugating enzyme E2 | P62068 | -2 |  |  |  |  |
| comp176168_c1_seq1|m.338078 | Ubiquitin-conjugating enzyme E2 | Q8N2K1 | -2 |  |  |  |  |
| comp174337_c0_seq1|m.302921 | Ubiquitin-conjugating enzyme E2 | K1QM65 | 2 |  |  |  |  |
| comp171350_c1_seq3|m.251857 | Ubiquitin-conjugating enzyme E2 | Q66KB0 |  | -2 | -2 |  |  |
| comp180445_c5_seq3|m.441527 | Ubiquitin carboxyl-terminal hydrolase | P62068 | -2 |  |  |  |  |
| comp180457_c1_seq1|m.441872 | Ubiquitin carboxyl-terminal hydrolase | Q9UPU5 | -2 |  |  |  |  |
| comp178573_c2_seq2|m.394665 | E3 ubiquitin-protein ligase | O60337 | -2 |  |  |  |  |
| comp172109_c0_seq2|m.264404 | Ubiquitin domain-containing protein | Q6PGH0 | -2 |  |  |  |  |
| comp121913_c0_seq1|m.89003 | Ubiquitin-40S ribosomal protein | P79781 | 2 |  |  |  |  |
| comp158519_c0_seq18|m.149444 | Ubiquitin | H8YJF5 | 5 | -8 | -8 |  |  |
| comp162332_c0_seq1|m.164546 | Ubiquitin C | A0A076KW18 |  | 9 |  |  |  |
| comp179594_c0_seq5|m.419755 | Ubiquitin ligase 2 | Q0R0E3 |  |  | 2 |  |  |
| comp173908_c0_seq3|m.294018 | Calreticulin | P27797 | 3 | 2 |  |  |  |
| comp159710_c0_seq1|m.153569 | Translocon-associated protein subunit delta | Q2TBX5 | 2 |  |  |  |  |
| comp164337_c0_seq1|m.176279 | Protein disulfide-isomerase | T2MGS0 | 3 |  |  |  |  |
| comp152707_c0_seq1|m.134644 | Protein disulfide-isomerase | A7YXX4 |  | -6 | -6 |  |  |
| comp181529_c0_seq1|m.472062 | cyclophilin-type (facilitate protein folding) | Q13356 | -3 |  |  |  |  |
| comp77110_c0_seq1|m.44113 | cyclophilin-type (facilitate protein folding) | Q9TW32 |  | -8 | -8 |  |  |
| comp178304_c0_seq5|m.388101 | permease (protein transport) | Q9Z2J0 | -3 | -4 | -3 |  |  |
| comp170060_c1_seq2|m.233549 | Calumenin | V9PBR3 |  | 5 | 2 |  |  |
| comp175025_c0_seq1|m.316220 | Hypoxia up-regulated protein 1 | Q63617 | 2 | 3 | 2 |  |  |
| comp179486_c0_seq1|m.416677 | Hypoxia up-regulated protein 1 | A0A076FS22 |  |  | 2 |  |  |
| comp182082_c1_seq1|m.487475 | Prefoldin | G3HLQ3 | 2 |  |  |  |  |
| comp174008_c1_seq3|m.295860 | ER unfolded protein response (GO) | Q9BQ90 | -4 |  |  |  |  |
| comp175787_c1_seq6|m.330406 | ER unfolded protein response (GO) | Q14703 | -3 |  |  |  |  |
| **Apoptosis** |  |  |  |  |  |  |  |
| comp174606_c1_seq2|m.308526 | Caspase | G8XQY3 | -2 |  |  |  |  |
| comp163741_c0_seq1|m.171954 | bcl (antiapoptotic) | B7SB93 |  |  | 3 |  |  |
| comp171230_c1_seq1|m.249816 | bax (proapoptotic) | B7SB94 | -2 |  |  |  |  |
| comp181103_c1_seq1|m.459527 | TNF receptor | T2M357 | -4 |  |  |  |  |
| comp181351_c1_seq1|m.466807 | Autophagy | Q96BY7 | -3 |  |  |  |  |
| comp166678_c1_seq1|m.195086 | Fas apoptotic inhibitory molecule | Q9NVQ4 | -4 |  |  |  |  |
| comp178483_c2_seq2|m.392596 | Mitochondrial pyruvate carrier 1 (Apoptosis-regulating basic protein) | P63031 | -2 | -3 |  |  |  |
| comp167562_c0_seq2|m.204346 | Programmed cell death 6 | Q7T3D5 | -2 |  |  |  |  |
| **Cell cycle** |  |  |  |  |  |  |  |
| comp178715_c2_seq1|m.397790 | Cell cycle control protein | T2M525 | -3 |  |  |  |  |
| **Cytoskeleton and extracellular skeleton** |  |  |  |  |  |  |  |
| comp169359_c0_seq1|m.224355 | Alpha-centractin | P61163 | -3 |  |  |  |  |
| comp180075_c0_seq4|m.431899 | Actin-related protein 2 | P61160 | -2 |  |  |  |  |
| comp171560_c1_seq3|m.255714 | Actin-related protein 10 | A7S805 | -2 |  |  |  |  |
| comp166131_c2_seq1|m.189552 | Actin, cytoplasmic 1 | P60709 | 2 |  |  |  | 3 |
| comp178314_c1_seq1|m.388355 | Actin, cytoskeletal 3 | Q25379 |  |  | 2 |  |  |
| comp161983_c2_seq3|m.162675 | Gelsolin-like protein 1 (Actin-modulator) | Q7JQD3 |  | -2 | -2 |  |  |
| comp177997_c2_seq5|m.381079 | Tubulin beta chain | P07437 |  | 7 |  |  |  |
| comp167606_c0_seq1|m.204795 | Tubulin beta chain | P07437 |  | -3 |  |  |  |
| comp172014_c0_seq1|m.262864 | Beta-tubulin | A0A060BJD1 |  | -6 | -6 | -5 |  |
| comp172956_c0_seq3|m.277948 | Tubulin alpha chain | P41351 |  | -3 | -3 |  |  |
| comp167332_c0_seq1|m.201877 | skeletal organic matrix protein 7 | B8WI85 | 2 | 2 | 2 |  |  |
| comp174212_c0_seq1|m.299272 | Insoluble matrix shell protein 1 | P86982 |  | 44 | 9 |  |  |
| comp178152_c0_seq3|m.384537 | Reversion-inducing cysteine-rich protein | O95980 | -4 |  |  |  |  |
| comp157526_c1_seq3|m.146372 | Cysteine-rich protein 1 | P50238 |  | -5 |  |  |  |
| comp177184_c0_seq2|m.360686 | Cysteine-rich protein | C0KTQ3 |  | 49 | 40 |  |  |
| comp159184_c0_seq1|m.151806 | Cysteine-rich protein 1 | P63255 | 2 |  |  |  |  |
| comp157136_c0_seq2|m.145341 | Mannose-binding lectin | B3V946 | 9 | -2 |  |  | 5 |
| comp159083_c0_seq1|m.151484 | Nematogalectin-related | E0D8Q5 |  |  |  | -4 |  |
| comp154345_c0_seq1|m.138304 | Collagen triple helix repeat-containing protein 1 | Q8CG08 | -10 | -65 | -40 |  |  |
| comp170631_c0_seq1|m.240974 | Collagen triple helix repeat-containing protein 1 | Q96CG8 |  | -4 |  |  |  |
| comp171210_c1_seq4|m.249562 | Collagen triple helix repeat-containing protein 1 | Q8CG08 |  | 16 |  |  |  |
| comp179682_c1_seq1|m.421761 | Collagen alpha-1(IV) chain | P02463 | -2 |  |  |  |  |
| comp179542_c2_seq3|m.418215 | Procollagen galactosyltransferase 1 | A5PK45 |  | 4 |  |  |  |
| comp181221_c2_seq5|m.463438 | Peroxidasin | V9GVF2 |  | 3 | 2 |  |  |
| comp178842_c0_seq1|m.401084 | Protocadherin-like | B8V7Q1 | -2 |  |  |  |  |
| comp174222_c0_seq10|m.299570 | Carbonic anhydrase | Q5ENU2 | -5 | -7 | -7 | -3 |  |
| comp177162_c2_seq1|m.360024 | Integrin alpha 1 | B2XW55 | -3 |  | 4 |  |  |
| comp172270_c1_seq1|m.266929 | Disintegrin and metalloproteinase domain-containing protein 10 | O14672 | -2 |  | 3 |  |  |
| comp68857_c0_seq1|m.27063 | Profilin-A | P22271 |  | 14 |  |  |  |
| comp178125_c0_seq1|m.383803 | Myosin-10 | Q61879 | -3 |  |  |  |  |
| comp180804_c0_seq1|m.451573 | Myosin V | Q9NBH3 | -3 |  |  |  |  |
| comp181109_c0_seq1|m.459706 | Myosin regulatory light chain-like protein | L7VNV4 |  | 5 | 3 |  |  |
| comp69077_c0_seq1|m.27647 | Dynein light chain roadblock-type 2 | S8GCV3 | 5 |  |  |  |  |
| **Calcium homeostasis** |  |  |  |  |  |  |  |
| comp161496_c1_seq2|m.160374 | Calmodulin | P62203 | 3 |  |  |  |  |
| comp181667_c2_seq2|m.476134 | Calmodulin, striated muscleCCM1 | P02597 | -5 | -3 |  |  |  |
| comp172120_c2_seq4|m.264642 | Calmodulin | P04352 |  | -4 |  |  |  |
| comp153900_c0_seq1|m.137280 | Calmodulin | P27165 |  | -50 | -43 |  |  |
| comp159234_c0_seq1|m.151931 | Calmodulin | O97341 |  |  | -2 |  |  |
| comp170066_c0_seq1|m.233722 | Calmodulin | Q9U6D3 |  |  | -2 |  |  |
| comp176581_c0_seq1|m.346817 | Calpain-5 | O15484 | -3 |  |  |  |  |
| **Carbon matabolism** |  |  |  |  |  |  |  |
| comp176586_c0_seq7|m.347075 | 2-oxoglutarate dehydrogenase, mitochondrial | Q02218 | -3 |  |  |  |  |
| comp175093_c4_seq1|m.317299 | Glyceraldehyde-3-phosphate dehydrogenase | Q4JHJ4 | 7 |  |  |  |  |
| comp172373_c0_seq2|m.268507 | Glyceraldehyde-3-phosphate dehydrogenase | K8YP56 |  |  | -11 |  |  |
| comp175445_c0_seq5|m.323893 | Phosphoenolpyruvate carboxykinase | P35558 |  |  | 5 |  |  |
| comp178473_c0_seq3|m.392401 | Protein phosphatase 1A | P35813 | -2 |  |  |  |  |
| **Lipid metabolism** |  |  |  |  |  |  |  |
| comp179678_c0_seq2|m.421706 | Plasma glutamate carboxypeptidase | Q9Y646 | -2 |  |  |  |  |
| comp173432_c1_seq3|m.285843 | phospholipid catabolic process (Phosphoinositide phospholipase C ) | H9ITM0 | -4 |  |  |  |  |
| comp175071_c1_seq2|m.316948 | phospholipid catabolic process (N-acyl-phosphatidylethanolamine-hydrolyzing phospholipase D) | Q769K2 | -2 |  |  |  |  |
| comp181156_c1_seq3|m.461463 | phospholipid catabolic process (Isocitrate dehydrogenase) | O75874 | -2 |  |  |  |  |
| comp167081_c0_seq1|m.199437 | phospholipid catabolic process (Lipase) | A7S6G4 |  | 4 |  |  |  |
| comp179373_c0_seq4|m.414305 | lipid catabolic process (Lipase) | T1IVL3 |  | 3 | 12 |  |  |
| comp171735_c0_seq1|m.257978 | phospholipid catabolic process | A7SL62 |  | 2 |  |  |  |
| comp170636_c2_seq1|m.241072 | peroxisome organization (Peroxisomal biogenesis factor 3) | A6H7C2 | -4 |  |  |  |  |
| comp169837_c1_seq3|m.230697 | peroxisome organization (Non-specific lipid-transfer protein) | P22307 | -2 |  |  |  |  |
| comp173143_c0_seq1|m.280975 | peroxisome organization (Lon protease homolog 2) | Q86WA8 |  |  | 2 |  |  |
| comp177818_c1_seq2|m.376468 | lipid transport (Oxysterol-binding protein-related protein 9) | Q96SU4 | -2 |  |  |  |  |
| comp178715_c2_seq2|m.397794 | lipid transport (Oxysterol-binding protein) | H2MSC3 | -2 |  |  |  |  |
| comp177818_c1_seq4|m.376481 | lipid transport (Oxysterol-binding protein-related protein 9) | Q96SU4 | -2 |  |  |  |  |
| comp155108_c0_seq3|m.140093 | Phospholipase A(2) | A7S6H4 |  | -4 |  |  |  |
| comp172027_c1_seq3|m.263114 | Cytosolic phospholipase A2 | K1P6P4 |  | 5 | 4 |  |  |
| **Cellular energy** |  |  |  |  |  |  |  |
| comp88799_c0_seq1|m.57506 | Mitochondrial ATP synthase (GO) | I1GBV0 | 2 |  |  |  |  |
| comp171805_c4_seq1|m.259266 | Mitochondrial ATP synthase (GO) | A7SY16 | 2 |  |  |  |  |
| comp104864_c0_seq1|m.71959 | Mitochondrial ATP synthase (GO) | E8Z6Q3 | 5 |  |  |  |  |
| comp168606_c0_seq13|m.215945 | ATP synthase subunit e, mitochondrial | P29419 | 2 |  |  |  |  |
| comp120241_c0_seq1|m.87079 | NADH dehydrogenase [ubiquinone] 1 alpha subcomplex subunit 5 | Q9CPP6 | 2 |  |  |  |  |
| comp159684_c0_seq1|m.153492 | NADH dehydrogenase [ubiquinone] iron-sulfur protein 4, mitochondrial | Q02375 | 2 |  |  |  |  |
| comp166488_c0_seq1|m.193288 | mitochondrial electron transport chain (NADH dehydrogenase [ubiquinone] flavoprotein 2, mitochondrial) | P19404 | 2 |  |  |  |  |
| comp159684_c0_seq1|m.153492 | mitochondrial electron transport chain | Q02375 | 2 |  |  |  |  |
| comp179506_c0_seq1|m.417282 | mitochondrial electron transport chain (Cytochrome b-c1 complex subunit 7) | A7SGK8 | 2 |  |  |  |  |
| comp146481_c0_seq1|m.123328 | mitochondrial electron transport chain (Cytochrome b-c1 complex subunit 9) | K4FS94 | 2 |  |  |  |  |
| **Cytochromes** |  |  |  |  |  |  |  |
| comp148056_c0_seq1|m.125468 | Cytochrome c | A0A061RYV8 | 8 |  |  |  | 3 |
| comp148056_c0_seq1|m.125468 | Cytochrome c | A0A061RYV8 |  |  |  | 2 |  |
| comp182383_c0_seq3|m.495764 | Cytochrome c oxidase subunit | Q62425 | -2 |  |  |  |  |
| comp156116_c0_seq1|m.142758 | Cytochrome c oxidase subunit 6A, mitochondrial | H9G6R7 | 2 |  |  |  |  |
| comp125041_c0_seq2|m.92977 | Cytochrome b5 domain-containing protein 1 | Q567I9 | 3 |  |  |  |  |
| comp176396_c1_seq3|m.342894 | Cytochrome P450 | B4YFB4 |  |  | 4 |  |  |
| **Cellular transport** |  |  |  |  |  |  |  |
| comp180786_c3_seq2|m.451245 | Ras GTPase-activating-like protein | T2MFN7 | -3 |  |  |  |  |
| comp158032_c0_seq1|m.147851 | GTP-binding nuclear protein Ran | P42558 | 6 |  |  |  |  |
| comp161853_c0_seq1|m.161978 | ion transport | Q13557 | -4 |  |  |  |  |
| comp173223_c0_seq1|m.282247 | ion transport | B3EWZ9 | -3 |  |  |  |  |
| comp176085_c0_seq1|m.336466 | ion transport | A7SVE8 | -3 |  |  |  |  |
| comp160394_c1_seq1|m.156070 | ion transport | Q08209 | -3 |  |  |  |  |
| comp173420_c1_seq1|m.285637 | ion transport | A7RGD8 | -3 |  |  |  |  |
| comp179990_c0_seq6|m.429739 | ion transport | Q9Y376 | -2 |  |  |  |  |
| comp173765_c3_seq16|m.291305 | ion transport | C3XUD0 |  | -4 |  |  |  |
| comp180167_c0_seq6|m.434095 | Sodium/potassium-transporting ATPase subunit alpha | P13607 | -4 |  |  |  |  |
| comp178125_c1_seq1|m.383849 | Sodium/potassium-transporting ATPase subunit beta-1-interacting protein 1 | Q4KMZ8 | -2 |  |  |  |  |
| comp143412_c0_seq1|m.117703 | Phosphate transport protein | B8BVT0 |  | -4 | -4 |  |  |
| comp173214_c0_seq2|m.282171 | ATP-binding cassette sub-family F member 2 | Q9UG63 |  |  | 4 |  |  |
| comp179660_c0_seq3|m.421324 | vesicle transport (Exocyst complex component 4) | Q62824 | -3 |  |  |  |  |
| **RNA** |  |  |  |  |  |  |  |
| comp182403_c0_seq1|m.496271 | splicing factor (Pre-mRNA-splicing factor syf2) | Q28G05 | -2 |  |  |  |  |
| comp179276_c2_seq6|m.411979 | splicing factor (Poly(U)-binding-splicing factor ) | Q5R469 | -2 |  |  |  |  |
| **DNA damage** |  |  |  |  |  |  |  |
| comp163284_c0_seq6|m.169317 | MutY homolog | Q8R5G2 | -3 |  |  |  |  |
| **Histones** |  |  |  |  |  |  |  |
| comp180979_c1_seq1|m.456322 | Core histone macro-H2A | Q1RLR9 | -3 |  |  |  |  |
| comp171695_c0_seq5|m.257467 | Histone H2A | I3KZV4 |  | -2 |  |  |  |
| comp171695_c0_seq3|m.257455 | Histone H2B 1/2 | Q5BJA5 |  | -2 |  | -2 |  |
| comp182703_c0_seq1|m.503377 | Putative histone 2B | E3UGH3 |  | -7 | -7 |  |  |
| **Innate immune response** |  |  |  |  |  |  |  |
| comp181211_c0_seq6|m.463104 | innate immune response (GO) | 0 | -6 |  |  |  |  |
| comp178557_c0_seq1|m.394318 | innate immune response (GO) | P17252 | -6 |  |  |  |  |
| comp181211_c0_seq2|m.463074 | innate immune response (GO) | P41240 | -4 |  |  |  |  |
| comp180720_c0_seq1|m.449029 | innate immune response (GO) | O75582 | -4 |  |  |  |  |
| comp161798_c0_seq1|m.161770 | innate immune response (GO) | P25098 | -3 |  |  |  |  |
| comp179179_c1_seq2|m.409564 | innate immune response (GO) | P62993 | -3 |  |  |  |  |
| comp160394_c1_seq1|m.156070 | innate immune response (GO) | Q08209 | -3 |  |  |  |  |
| comp179179_c1_seq5|m.409576 | innate immune response (GO) | P62993 | -3 |  |  |  |  |
| comp173418_c0_seq10|m.285586 | innate immune response (GO) | A7SLZ2 | -3 |  |  |  |  |
| comp180075_c0_seq4|m.431899 | innate immune response (GO) | P61160 | -2 |  |  |  |  |
| comp169648_c0_seq2|m.228353 | innate immune response (GO) | P62195 | -2 |  |  |  |  |
| comp180075_c0_seq5|m.431907 | innate immune response (GO) | P61160 | -2 |  |  |  |  |
| comp164681_c1_seq1|m.178742 | innate immune response (GO) | P52564 | -2 |  |  |  |  |
| comp173418_c0_seq16|m.285608 | innate immune response (GO) | A7SLZ2 | -2 |  |  |  |  |
| comp161139_c0_seq1|m.159071 | innate immune response (GO) | P63000 | -2 |  |  |  |  |
| comp169648_c0_seq1|m.228347 | innate immune response (GO) | P62195 | -2 |  |  |  |  |
| comp172496_c0_seq1|m.270470 | innate immune response (GO) | P62993 | -2 |  |  |  |  |
| comp175366_c0_seq1|m.322587 | innate immune response (GO) | P12931 | -2 |  |  |  |  |
| comp178679_c0_seq1|m.397108 | innate immune response (GO) | P49137 | -2 |  |  |  |  |
| comp154990_c0_seq1|m.139791 | innate immune response (GO) | P62333 | 2 |  |  |  |  |
| comp121913_c0_seq1|m.89003 | innate immune response (GO) | P79781 | 2 |  |  |  |  |
| comp166131_c2_seq1|m.189552 | innate immune response (GO) | P60709 | 2 |  |  |  |  |
| comp177737_c0_seq7|m.374280 | innate immune response (GO) | P28066 | 2 |  |  |  |  |
| comp149471_c0_seq1|m.128159 | innate immune response (GO) | P25786 | 2 |  |  |  |  |
| comp163766_c0_seq2|m.172113 | innate immune response (GO) | P60709 | 4 |  |  |  |  |
| comp166392_c0_seq2|m.192363 | innate immune response (GO) | P51812 |  |  | 2 |  |  |
| **NOTCH signaling** |  |  |  |  |  |  |  |
| comp181274_c0_seq3|m.464856 | NOTCH signaling (GO) | G9JWI5 | -4 |  |  |  |  |
| comp169875_c0_seq1|m.231071 | NOTCH signaling (GO) | P31266 | -2 |  |  |  |  |
| comp173590_c0_seq3|m.288355 | NOTCH signaling (GO) | O60547 | -2 |  |  |  |  |
| **WNT signaling** |  |  |  |  |  |  |  |
| comp175092_c2_seq1|m.317234 | WNT signaling (GO) | Q9H694 | -5 |  | 2 |  |  |
| comp166759_c0_seq1|m.196041 | WNT signaling (GO) | O54949 | -4 |  |  |  |  |
| comp170415_c0_seq22|m.238433 | WNT signaling (GO) | P48729 | -3 |  |  |  |  |
| comp177803_c0_seq1|m.376143 | WNT signaling (GO) | P84022 | -3 |  |  |  |  |
| comp173800_c0_seq10|m.292051 | WNT signaling (GO) | P92208 | -3 |  |  |  |  |
| comp173800_c0_seq1|m.292011 | WNT signaling (GO) | P92208 | -3 |  |  |  |  |
| comp174442_c2_seq1|m.305793 | WNT signaling (GO) | Q5IHW1 | -3 |  |  |  |  |
| comp169875_c0_seq1|m.231071 | WNT signaling (GO) | P31266 | -2 |  |  |  |  |
| comp169648_c0_seq2|m.228353 | WNT signaling (GO) | P62195 | -2 |  |  |  |  |
| comp173773_c3_seq3|m.291526 | WNT signaling (GO) | Q6P3W7 | -2 |  |  |  |  |
| comp166269_c0_seq1|m.191350 | WNT signaling (GO) | Q19QV6 | -2 |  |  |  |  |
| comp161139_c0_seq1|m.159071 | WNT signaling (GO) | P63000 | -2 |  |  |  |  |
| comp169648_c0_seq1|m.228347 | WNT signaling (GO) | P62195 | -2 |  |  |  |  |
| comp175366_c0_seq1|m.322587 | WNT signaling (GO) | P12931 | -2 |  |  |  |  |
| comp169933_c0_seq1|m.231939 | WNT signaling (GO) | A7SCF4 | -2 |  |  |  |  |
| comp163753_c0_seq1|m.172047 | WNT signaling (GO) | A7SFU2 | -2 |  |  |  |  |
| comp166269_c0_seq2|m.191358 | WNT signaling (GO) | Q19QV6 | -2 |  |  |  |  |
| comp178473_c0_seq3|m.392401 | WNT signaling (GO) | P35813 | -2 |  |  |  |  |
| comp154990_c0_seq1|m.139791 | WNT signaling (GO) | P62333 | 2 |  |  |  |  |
| comp177737_c0_seq7|m.374280 | WNT signaling (GO) | P28066 | 2 |  |  |  |  |
| comp149471_c0_seq1|m.128159 | WNT signaling (GO) | P25786 | 2 |  |  |  |  |
| comp160884_c1_seq1|m.158095 | WNT signaling (GO) | P48148 |  | 2 | 6 |  |  |
| comp171279_c0_seq1|m.250626 | WNT signaling (GO) | Q5IHV8 |  | -2 | -2 |  |  |
| comp170926_c7_seq1|m.245006 | WNT signaling (GO) | H3DJS1 |  |  | -2 | -2 |  |

**Table S4.**

Number of genes in enriched GOs *in S. pistillata* unique DEGs after combined pH-temperature treatment (sty6pH unique) compared to temperature treatment (sty 6) at +6^0^C above control. GOs are that are related to cellular transport are marked in purple, protein localization in blue, proteolysis processes in pink and protein ubiquitination in yellow.

Table S5.


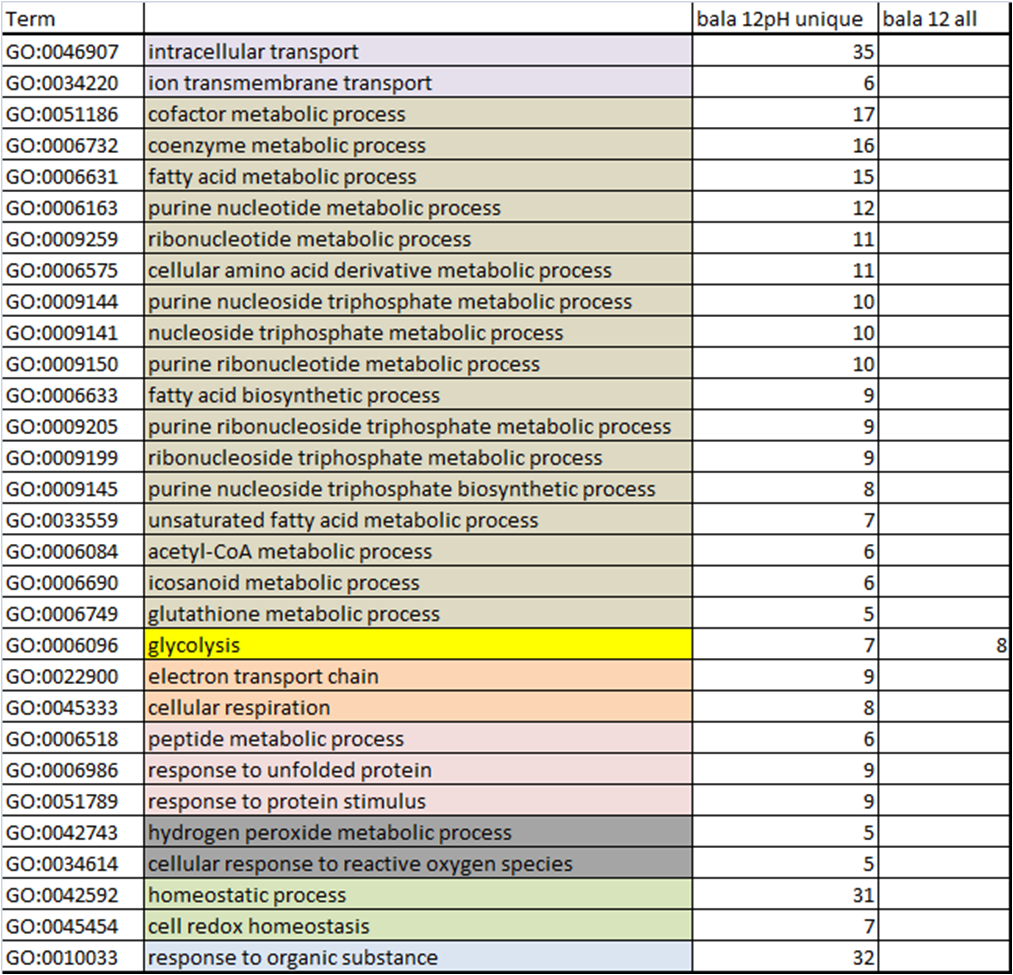
Number of genes in enriched GOs in *B. europaea* unique DEGs after combined pH-temperature treatment (bala12pH unique) compared to temperature treatment (bala12) at +12^0^C above control. GOs that are related to cellular transport are marked in purple, metabolism processes in brown, glycolysis in yellow, cellular respiration processes in orange, protein modifications processes in pink, oxidative stress processes in grey, homeostasis in green and other in blue.

**Table S6**

Primers and PCR conditions for qRT-PCR validations

References

1. Ulitsky, I. *et al.* Expander: from expression microarrays to networks and functions. *Nat. Protoc.* **5,** 303–22 (2010).

2. Zhi-Liang, H., Bao, J. & James M., R. A web-based program to batch gene ontology classification categories. *Online J. Bioinforma.* **9.2,** 108–112 (2008).

3. Crooks, G. E., Hon, G., Chandonia, J. & Brenner, S. E. WebLogo: a sequence logo generator. *Genome Res* **14,** 1188–1190 (2004).
